# The AUREX cell: a versatile *operando* electrochemical cell for studying catalytic materials using X-ray diffraction, total scattering and X-ray absorption spectroscopy under working conditions

**DOI:** 10.1107/S1600576724007817

**Published:** 2024-09-20

**Authors:** Sara Frank, Marcel Ceccato, Henrik S. Jeppesen, Melissa J. Marks, Mads L. N. Nielsen, Ronghui Lu, Jens Jakob Gammelgaard, Jonathan Quinson, Ruchi Sharma, Julie S. Jensen, Sara Hjelme, Cecilie Friberg Klysner, Simon J. L. Billinge, Justus Just, Frederik H. Gjørup, Jacopo Catalano, Nina Lock

**Affiliations:** a Department of Biological and Chemical Engineering Aarhus University Åbogade 40 8200Aarhus N Denmark; b Deutsches Elektronen-Synchrotron (DESY) Notkestrasse 85 22607Hamburg Germany; c Interdisciplinary Nanoscience Center (iNANO) Aarhus University Gustav Wieds Vej 14 8000Aarhus C Denmark; d Carbon Dioxide Activation Center (CADIAC), Department of Biological and Chemical Engineering Aarhus University Åbogade 40 8200Aarhus N Denmark; e Department of Applied Physics and Applied Mathematics Columbia University New York NY10027 USA; f MAX IV Laboratory Lund University Fotongatan 2 221 00Lund Sweden; g Department of Chemistry Aarhus University Langelandsgade 8000Aarhus Denmark; The University of Western Australia, Australia

**Keywords:** *operando* studies, cell design, X-ray scattering, X-ray absorption spectroscopy, structure–property relationships, electrocatalysis

## Abstract

Understanding the structure–property relationship in electrocatalysts under working conditions is crucial for the rational design of novel and improved catalytic materials. This paper presents the Aarhus University reactor for electrochemical studies using X-rays (AUREX) *operando* electrocatalytic flow cell, designed as an easy-to-use versatile setup with a minimal background contribution and a uniform flow field to limit concentration polarization and handle gas formation. The cell has been employed to measure *operando* total scattering, diffraction and absorption spectroscopy as well as simultaneous combinations thereof on a commercial silver electrocatalyst for proof of concept. This combination of *operando* techniques allows for monitoring of the short-, medium- and long-range structure under working conditions, including an applied potential, liquid electrolyte and local reaction environment. The structural transformations of the Ag electrocatalyst are monitored with non-negative matrix factorization, linear combination analysis, the Pearson correlation coefficient matrix, and refinements in both real and reciprocal space. Upon application of an oxidative potential in an Ar-saturated aqueous 0.1 *M* KHCO_3_/K_2_CO_3_ electrolyte, the face-centered cubic (f.c.c.) Ag gradually transforms first to a trigonal Ag_2_CO_3_ phase, followed by the formation of a monoclinic Ag_2_CO_3_ phase. A reducing potential immediately reverts the structure to the Ag (f.c.c.) phase. Following the electrochemical-reaction-induced phase transitions is of fundamental interest and necessary for understanding and improving the stability of electrocatalysts, and the *operando* cell proves a versatile setup for probing this. In addition, it is demonstrated that, when studying electrochemical reactions, a high energy or short exposure time is needed to circumvent beam-induced effects.

## Abstract 2

The AUREX *operando* electrocatalytic cell is demonstrated to be a versatile setup for elucidating structure–property relations by means of X-ray total scattering and multimodal absorption and diffraction techniques using an Ag electrocatalyst for proof of concept.

## 1. Introduction

There is increased interest in electrochemical energy conversion technologies, *e.g.* through electrolysis, to mitigate the increasing CO_2_ concentrations in the atmosphere (Hori, 2008 ▸). The rational design of novel and improved materials for such technologies requires a fundamental understanding of the electrocatalyst structure. Beyond understanding the structural characteristics of the pristine catalyst, characterizing the structural evolution under working conditions is essential for enhancing their performance by design (Jiang *et al.*, 2018 ▸). The catalysts will often undergo structural changes during electrocatalytic processes, rendering the structural characteristics of the pristine material largely irrelevant (Li *et al.*, 2017 ▸; Zhu *et al.*, 2019 ▸). For example, short-term exposure to working conditions can lead to an increase in performance due to an activation of the catalyst or, conversely, long-term exposure can lead to degradation of the catalyst (Frank *et al.*, 2021 ▸, 2024 ▸). However, despite the importance of understanding the transient structural behavior, including the formation of the actual active phase and the fundamental processes occurring at the electrode, such factors are seldom studied and rarely understood (Zhu *et al.*, 2021 ▸).

*Ex situ* and *postmortem* characterization of catalysts are common. Yet, they are inadequate in describing the transient behavior of the catalyst when subjected to operating conditions such as an applied electrical potential, contact with the electrolyte and exposure to the local reaction environment, especially when a relaxation of reactive intermediates occurs.

An increasing number of *operando* studies during electrocatalysis have been published in recent years, offering some insight into the evolution of the catalytic system under operating conditions. Different characterization techniques have been utilized, *e.g.* microscopy, spectroscopy and scattering (Li & Gong, 2020 ▸). In particular, combining *operando* electrochemical studies with high-energy synchrotron X-ray techniques offers the ability to obtain transient structural insights, as high-energy X-rays can penetrate through an entire electrochemical cell, including the electrolyte liquids (Magnussen *et al.*, 2024 ▸; Baggio & Grunder, 2021 ▸). However, achieving high signal-to-noise ratios is still challenging as the catalyst loading is typically low. The signal from a catalytic thin film is typically several orders of magnitude weaker than that from bulk crystals, making synchrotron X-rays preferable to conventional laboratory X-rays to achieve a sufficiently intense beam (Magnussen *et al.*, 2024 ▸).

X-ray absorption spectroscopy (XAS) has been widely utilized in *operando* electrochemical studies due to its element-specific information and the possibility to probe the oxidation state of metal atoms, coordination numbers and local environment (Abbott *et al.*, 2016 ▸; Lassalle-Kaiser *et al.*, 2017 ▸; Pedersen *et al.*, 2018 ▸; Firet *et al.*, 2019 ▸, 2020 ▸; Wu, Guo *et al.*, 2021 ▸). In XAS experiments, tens of minutes are conventionally needed to acquire a single spectrum, which is not suitable for time-resolved *operando* experiments. Yet, the time resolution of XAS experiments at specialized beamlines has increased to minutes (Wu, Guo *et al.*, 2021 ▸) and even sub-seconds, which makes it possible to study dynamic electrochemical reaction conditions (Timoshenko *et al.*, 2022 ▸).

For structural characterization of crystalline catalysts, X-ray diffraction (XRD) is the most suitable technique, as accurate crystallographic parameters can be obtained under transformations using fast and easily accessible instrumentation (Dionigi *et al.*, 2020 ▸; Moss *et al.*, 2023 ▸; Qiao *et al.*, 2023 ▸). However, XRD provides very little information on disordered, nanostructured or amorphous contents.

Examples exist of electrochemical *operando* cells for both X-ray spectroscopy and diffraction, allowing both crystalline and amorphous phases to be probed (Farmand *et al.*, 2019 ▸; Timoshenko *et al.*, 2022 ▸). However, the local structural information achievable with XAS under *operando* conditions is usually limited to the first coordination shells, approximately up to 6 Å.

Total scattering and pair distribution function (PDF) analysis can provide longer-range information than XAS and is not limited to crystalline catalysts, as is the case for XRD. In contrast to conventional XRD, total scattering includes both Bragg and diffuse scattering, which is measured over a wide range of reciprocal space. This allows for the recovery of information on the local structure and amorphous phases. While the diffuse signal is regarded as a part of the background in XRD analysis, it contains important information for PDF analysis (Billinge & Kanatzidis, 2004 ▸; Billinge & Levin, 2007 ▸). Thus, the experimental background needs to be recorded with high accuracy, such that the contributions from air scattering, the sample environment, including the sample container and liquid electrolyte, Compton scattering *etc.* can be subtracted (Diaz-Lopez *et al.*, 2020 ▸). As this is a challenge for the complex sample environment in an electrochemical setup, only a few studies have utilized PDF analysis of electrocatalysts under *operando* conditions. Examples include the investigation of the dynamic behavior of nano­alloys as the cathode of a proton exchange membrane fuel cell (Petkov *et al.*, 2019 ▸; Kong *et al.*, 2020 ▸; Wu, Caracciolo *et al.*, 2021 ▸). In addition, amorphous thin-film catalysts have been characterized in microfluidic (Kwon *et al.*, 2019 ▸) and multielectrode (Kwon *et al.*, 2023 ▸) electrochemical cells, and, most recently, the changes in <3 nm small iridium nanoparticles have been tracked with PDF and small-angle X-ray scattering analysis during the acidic oxygen evolution reaction (OER) (Pittkowski *et al.*, 2023 ▸).

Ideally, a combination of all the above techniques should be utilized as it is necessary to examine both the local atomic environment and the long-range order, to obtain a complete understanding of the catalyst (van der Stam, 2023 ▸; Zhu *et al.*, 2021 ▸). However, due to the different technical requirements for data collection using each technique, as well as the diverse expertise required for analysis of the data, to the best of our knowledge, no single *operando* cell has yet been reported that is suitable for all the techniques. This study addresses this gap, presenting the design of the Aarhus University reactor for electrochemical studies using X-rays (AUREX) *operando* electrochemical flow cell, and demonstrates its features as a versatile setup for time-resolved *operando* electrocatalysis studies. Its use for X-ray scattering and absorption experiments is here detailed, including total scattering and PDF analysis, as well as multimodal XAS and XRD.

By using a commercial Ag nanoparticle electrocatalyst, it is shown how a combination of the three structural characterization techniques together with the electrochemical traces offers unique insights into the stability of the electrode at both reductive (cathodic) and oxidative (anodic) potentials. Ag nanoparticle catalysts have great potential for large-scale applications due to their relatively low cost (compared with other precious metals used in electrocatalysis, such as Au, Pt and Ir), robustness, high material utilization and high selectivity towards the electrochemical CO_2_ reduction reaction (eCO_2_RR) to CO (Hori, 2008 ▸; Lu *et al.*, 2014 ▸; Kim *et al.*, 2015 ▸). Ag catalysts have previously been studied with *operando* XAS techniques under reductive potentials to reveal the presence of oxide species (Firet *et al.*, 2019 ▸) and defects (Wu, Guo *et al.*, 2021 ▸), and to elucidate crystallite sizes (Firet *et al.*, 2020 ▸). While Ag excels in the eCO_2_RR at reductive potentials, it is usually not a desired catalyst at oxidative potentials due to its limited stability and dissolution at high potentials (Linge *et al.*, 2023 ▸). However, the modification of Ag by applying an anodic potential can lower the required energy input, *i.e.* the overpotential for the eCO_2_RR, by forming oxide (Ma *et al.*, 2016 ▸) and carbonate (Ma *et al.*, 2018 ▸) phases (depending on the electrolyte). In addition, pulsed electrolysis, where the potential is switched between cathodic and anodic, is promising in improving the selectivity and stability of other eCO_2_RR catalysts (Timoshenko *et al.*, 2022 ▸; Obasanjo *et al.*, 2023 ▸). Thus, studying the Ag catalyst behavior at both reductive and oxidative potentials is of fundamental interest.

The *operando* experiments presented in this study allow for monitoring and refinement of the structure and its phase transitions, thereby elucidating the active phase. The oxidation and reduction processes are captured during cyclic voltammetry, along with the formation of two different Ag_2_CO_3_ phases at potentials more oxidative than the anodic peak potential and the immediate reduction to Ag at cathodic potentials. Such insights into the electrocatalyst degradation/dissolution mechanism are needed to understand and improve the stability. The excellent data quality and a fast time resolution of a few seconds for both scattering and XAS experiments allow for insights from model-free analysis techniques including multivariate component analysis such as non-negative matrix factorization (NMF), linear combination analysis (LCA) and the correlations with the Pearson correlation coefficient (PCC) matrix, as well as model-dependent analysis techniques including structural refinements of both PDF and XRD data. Thus, we demonstrate the versatility of the setup in elucidating the structure–property relations of electrocatalysts under operating conditions.

## 2. The AUREX *operando* cell design

The AUREX *operando* cell was designed to be easy to use, minimize the background, especially from the liquid electrolyte, obtain a high signal-to-noise ratio of the active catalyst layer and handle gas formation, *e.g.* produced during the electrocatalytic reactions and possibly radiolysis (X-ray-induced water splitting). The basic design concept and, in particular, the radial geometry and conical penetration was inspired and adapted from Argonne’s multi-purpose *in situ*X-ray (AMPIX) cell (Borkiewicz *et al.*, 2012 ▸). The key differences in the present setup in comparison with the AMPIX cell are that the half-cells are solid, electrically conductive, and machined to avoid sealings and reduce leaks. Additionally, the AUREX cell is composed of two identical symmetric half-cells, each containing a reference electrode. A fast cell assembly/disassembly at the synchrotron facility is possible due to wing nuts and PEEK (polyetheretherketone) finger-tight fittings for the inlet/outlet electrolyte streams and the reference electrodes.

**Figure 1:**
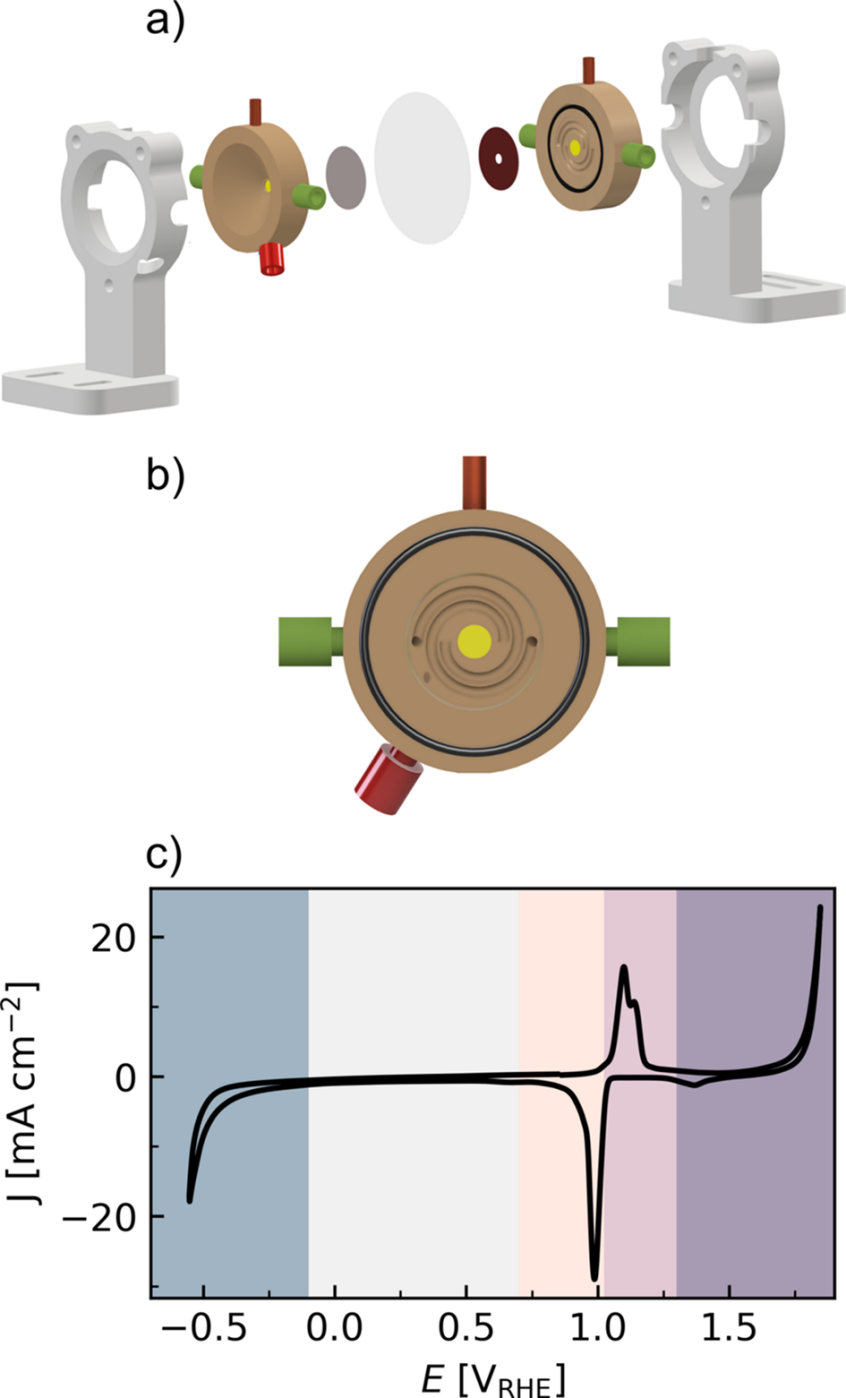
(*a*) Full cell assembly consisting of two PEEK backplates, two graphite half-cells, a working electrode, the membrane and a counter electrode. (*b*) Magnification of the half-cell with the interdigitated flow field, electrolyte inlet and outlet ports in green, the reference electrode port in red, and the Cu current collector at the top. (*c*) CV recorded in 0.1 *M* KHCO_3_/K_2_CO_3_ Ar-saturated electrolyte with a scan rate of 5 mV s^−1^. The shaded regions represent the region of eCO_2_RR/HER (in blue), the non-faradaic region (in gray), the redox reduction (pale orange) and oxidation (lavender), and the OER region (purple).

The body of the electrochemical *operando*X-ray flow cell is constructed of two graphite half-cells (grade RCGBPP01, RoyalEliteRoyCarbon, outer diameter 50 mm), compressed together via two PEEK back plates [Fig. 1 ▸(*a*)] and three stainless steel bolts. The external surface of the graphite is coated with a hydro­phobic thin layer (spray seal, Maston) to further decrease its gas/liquid permeability. To allow for the transmission of outgoing X-rays, the half-cells are machined with conical truncated holes leaving a window for the X-rays of 6 mm in diameter and a thickness of 0.2 mm. The cone opening is 110°. This minimizes blocking of the scattered X-rays, making it possible to measure high scattering angles and thereby a large portion of the reciprocal space (*e.g. Q* = 21 Å^−1^ in full ring configuration at P02.1 DESY with an X-ray energy of 60 keV), as well as not compromising the 90° required at most beamlines to measure XAS in fluorescence geometry with multielement detectors. In addition, the window area of 28 mm^2^ allows for probing multiple sample positions with a millimetre-to-micrometre-sized beam. Each half-cell has a central groove for hosting the electrode (diameter 25 mm, depth 200 µm) and a spiral interdigitated flow field [channel width 2 mm, depth 1 mm, Fig. 1 ▸(*b*)].

The half-cells are equipped with an inlet and outlet port (IDEX, 1/8″) for the electrolyte, which is pumped utilizing peristaltic pumps (REGLO ICC, ISMATEC) from the respective electrolyte reservoir. The half-cells have an additional port for a leakless reference electrode entering the side surface of the half-cell at an angle of 22°, allowing for the reference electrode to be in close contact with the working or counter electrodes. A low-resistance electrical coupling is ensured via copper plugs (4 mm diameter) which are connected to the leads of an electrochemical workstation. Disc-shaped electrodes with a 5.06 cm^2^ surface area are hosted in the graphite recesses, and the anodic and cathodic compartments are galvanically separated with an ion exchange membrane (thickness up to 100 µm) sealed with an O-ring (M-seals, internal diameter 39.45 mm, cross section 1.78 mm, material NBR70). The cell can be used to follow any catalyst system that can be supported (*e.g.* on carbon paper), making it a versatile setup.

The electrolyte flow (5–10 ml min^−1^) together with the interdigitated spiral flow field ensures the removal of potential gas bubbles formed (including H_2_ or O_2_) and provides continuous fresh electrolyte (including dissolved CO_2_ for eCO_2_RR studies) to the working and counter electrodes, thus partially mitigating the effect of concentration polarization from both electrochemical reactions and radiolysis of the liquid electrolyte. Additional uniformity of the flow field is ensured by the catalyst support, which in a liquid/gas or gas/gas configuration can also act as a gas diffusion layer. In contrast to the working electrode, the counter electrode features a hole in the X-ray beam path with the same diameter as the window, to avoid any contribution to the scattering or absorption signal.

From electrochemical impedance spectroscopy (EIS) (Section S1.1 in the supporting information, Figs. S1–S3), the ohmic resistance, which represents the electronic and ionic resistances of cables, current collectors, terminals, electrodes, electrolyte and membrane, can be estimated in the high-frequency region (*f* ∈ [10^2^;10^4^] Hz) as 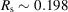
 Ω (∼1.00 Ω cm^2^). From the same data, the value of the internal cell resistance cannot be accurately measured since the data exhibit mass transfer limitation behavior. This is due to the zero-bias used in the EIS experiments, *i.e.* EIS was recorded in proximity to the open circuit potential. In any case, from the fitting of the EIS data (Section S1.1, supporting information) the internal resistance of the cell, including the charge transfer resistance, was estimated as *R*_in_ = 0.27 Ω (1.37 Ω cm^2^). *R*_in_ during working conditions can also be estimated from the voltage loss (*e.g.* iR drop) measured in the non-faradaic region recorded at the synchrotron facilities before the *operando* experiments. Depending on the experiments performed, *R*_in_ = 0.4–0.6 Ω (2.02–3.04 Ω cm^2^), which is indeed compatible with the results from EIS.

For the experiments performed in the present work, the following configuration has been adopted: the working electrode with the active catalyst consists of supported Ag nanoparticles with a nominal loading of 1 mg cm^−2^ on carbon paper (Dioxide materials) (Kutz *et al.*, 2017 ▸). The actual loading of the catalyst was determined by inductively coupled plasma–optical emission spectrometry (ICP–OES) to be 1.4 ± 0.1 mg cm^−2^. Carbon paper coated with an IrO_2_/ionomer mixture (Dioxide materials) (Kutz *et al.*, 2017 ▸) was utilized as the counter electrode, and a leak-free Ag/AgCl (saturated KCl) electrode (ElectroCell LF-1) was used as the reference electrode in the working electrode compartment. All experiments were performed in aqueous 0.1 *M* KHCO_3_/K_2_CO_3_ electrolyte buffer solution, saturated with either Ar (pH = 9.3) or CO_2_ (pH = 6.8). The cathodic and anodic chambers were separated by a Sustainion X37 membrane (Dioxide materials, thickness 50 µm). The choice of graphite for the half-cells ensures good electrical conductivity, although it contributes to significant Bragg scattering. Thus, even though the entire half-cell can be machined from one solid piece of graphite (to increase the bursting pressure of the cell), in this study a thin layer of Kapton (70 µm) for the cell window is chosen due to its amorphous nature, low scattering power and absorption of X-rays. This choice limits the maximum cell overpressure to a few tenths of a bar above atmosphere.

The *operando* cell was used in transmission geometry, which is the simplest geometry and possible when the catalyst is a thin film with a sufficiently high loading (Asset *et al.*, 2019 ▸). This makes the cell easy to use with little to no changes to the instrument or beamline configuration. Considering the above-mentioned design choice and cell configuration, the X-ray beam penetrates the two Kapton windows, the working electrode with the catalyst, the membrane, and the electrolyte on both sides.

Cyclic voltammetry (CV) shows the full electrochemical behavior of the Ag catalyst in the cell [Fig. 1 ▸(*c*)], distinguishing the different regions investigated in detail during the *operando* studies. From the most negative to the most positive potential, with all potentials reported with respect to the reversible hydrogen electrode (RHE), it is possible to observe the eCO_2_RR and the hydrogen evolution reaction (HER) [in the present work studied with total scattering/PDF analysis; see Fig. S5(*b*) for the electrocatalytic response in CO_2_ versus Ar-saturated electrolyte], the redox region of Ag (studied with total scattering/PDF analysis, XRD and XAS), and the OER (studied with total scattering/PDF analysis).

## 3. *Operando* electrocatalysis and redox chemistry of a commercial Ag catalyst

### 3.1. Total scattering and PDF

Often, intermediates or active phases are disordered, nanostructured or amorphous. In such cases, the structure can be elucidated with total scattering and PDF analysis. Most electrocatalytic *operando* cells are not optimized for PDF experiments, as the collection of data with sufficient quality requires a high energy covering a sufficiently large range of reciprocal space, as well as a challenging background-subtraction procedure.

The AUREX *operando* cell is demonstrated for total scattering measurements on a Ag catalyst with an X-ray energy of 60 keV at P02.1 at PETRA III, Germany (Dippel *et al.*, 2015 ▸). The potential was stepped to increasingly higher absolute values in CO_2_ and Ar-saturated electrolyte to emulate the active structure during the eCO_2_RR and the competing HER at negative potentials as well as the OER at positive potentials. In addition, the redox-reaction-induced phase transition during CV in the faradaic region around the redox peaks of Ag is investigated in detail. While the electrocatalytic reactions (eCO_2_RR, HER or OER) occur at the surface of the catalyst (interphase between electrolyte and catalyst), the oxidation/reduction of the metal might penetrate deeper than a monolayer (Wei *et al.*, 2019 ▸). Therefore, the two regions investigated in this work, *i.e.* above the onset potentials of electrocatalysis and the redox region, are likely to involve a different number of active metal atoms; thus a difference in the amount of active phase actually probed by the X-ray beam should be expected.

**Figure 2:**
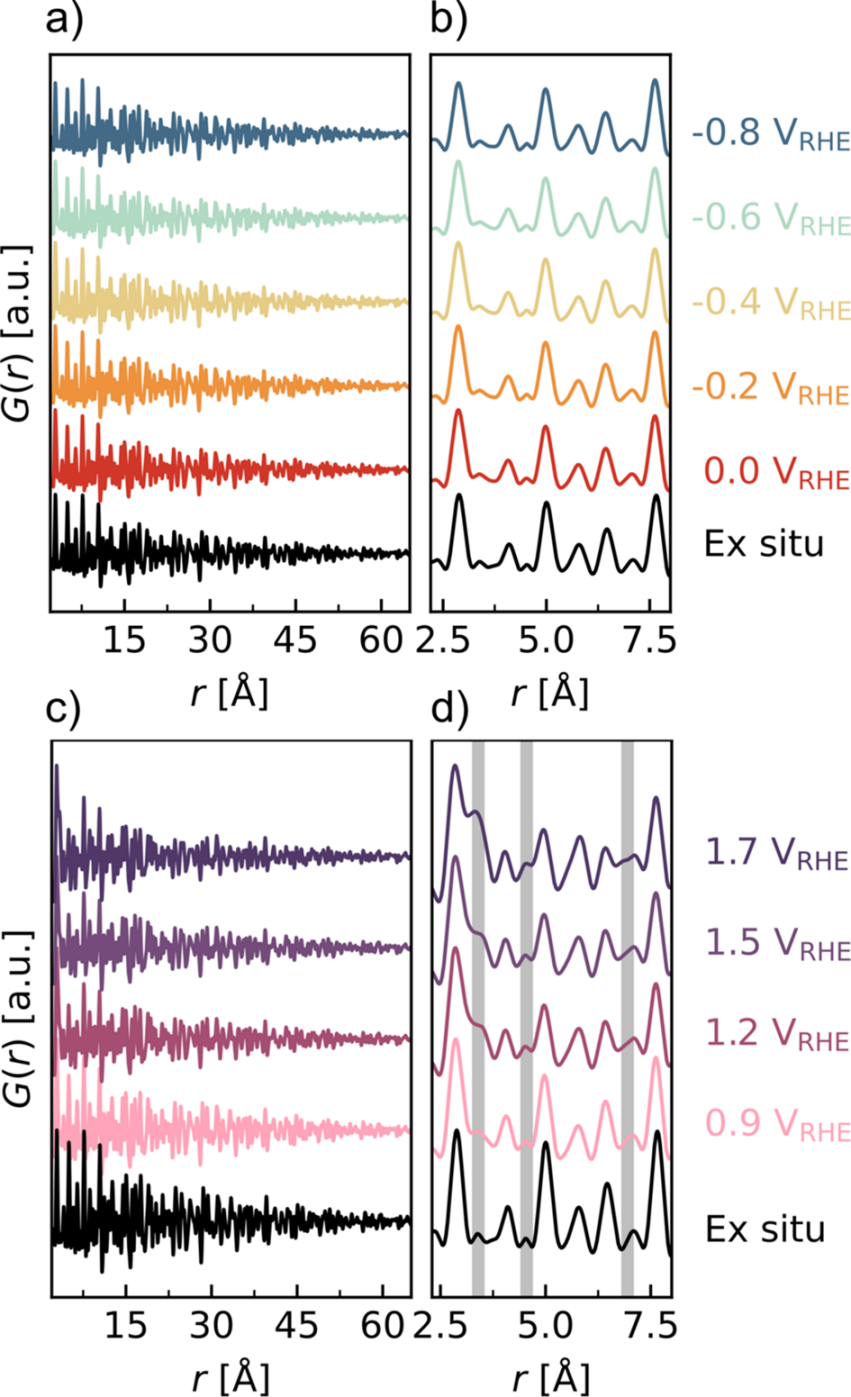
*Operando* PDF data collected during stepped chronoamperometry at increasingly high absolute values of the applied electrical potential (*a*)–(*b*) during the eCO_2_RR in a CO_2_-saturated aqueous 0.1 *M* KHCO_3_/K_2_CO_3_ electrolyte and (*c*)–(*d*) under oxidative potentials in an OER experiment in Ar-saturated electrolyte. (*a*) and (*c*) show the longer-range correlations while (*b*) and (*d*) are magnifications of the local regions. Gray lines in (*d*) highlight regions of structural changes. The PDF data are the last 180 s of each potential step summed (see the supporting information for details of the time voltage evolution).

The potential was stepped to increasingly negative potentials in CO_2_-saturated electrolyte until a significant eCO_2_RR current was recorded at −0.8 V_RHE_ [Fig. S6(*a*)]. The total scattering function *F*(*Q*) indicates a high similarity of the *operando* data to the catalyst measured *ex situ* with all Bragg reflections preserved in *Q* space; however, there is a slight increase in the diffuse signal, seen as an amorphous background [Fig. S6(*b*)]. The Fourier transform of *F*(*Q*) gives the PDF, here extended to 60 Å [Fig. 2 ▸(*a*)], and a magnification on the local range [Fig. 2 ▸(*b*)]. Only a few structural changes are observed locally, and refinements of the patterns (Fig. S7) corroborate this with a high similarity in the refined parameters (Table S1). An increase in disorder is observed by comparing data recorded under *operando* conditions with those of the *ex situ* catalyst (with increased thermal displacement parameters, Fig. S9), along with an apparent slight contraction of the unit-cell parameter *a* (on the fifth decimal). Interestingly, the size of the longest atomic pair distribution distance (as a measure of the particle size) increases to a larger value of 15.0 (1) nm at the most reducing potential of −0.8 V_RHE_, compared with 14.5 (1) nm for the *ex situ* catalyst. An increase in crystallite size and the contraction of the Ag—Ag bonds have also been observed in a recent *operando* XAS study (Firet *et al.*, 2020 ▸). In addition, growth in the size of Pt nanoparticle electrocatalysts has been observed (at both reductive and oxidative potentials) and attributed to catalyst dissolution and redeposition (Ostwald ripening) (Smith *et al.*, 2008 ▸) as well as nanoparticle aggregation and coalescence (Martens *et al.*, 2022 ▸). Thus, it is expected that similar mechanisms could take place on the surface of the Ag catalyst. The present experiments have probed the initial changes during operation; however, further restructuring might occur after long-term use, *e.g.* several hundreds of hours. The possible competing HER was investigated in Ar-saturated electrolyte, revealing similar structural changes due to the reducing potential (Fig. S8).

**Figure 3:**
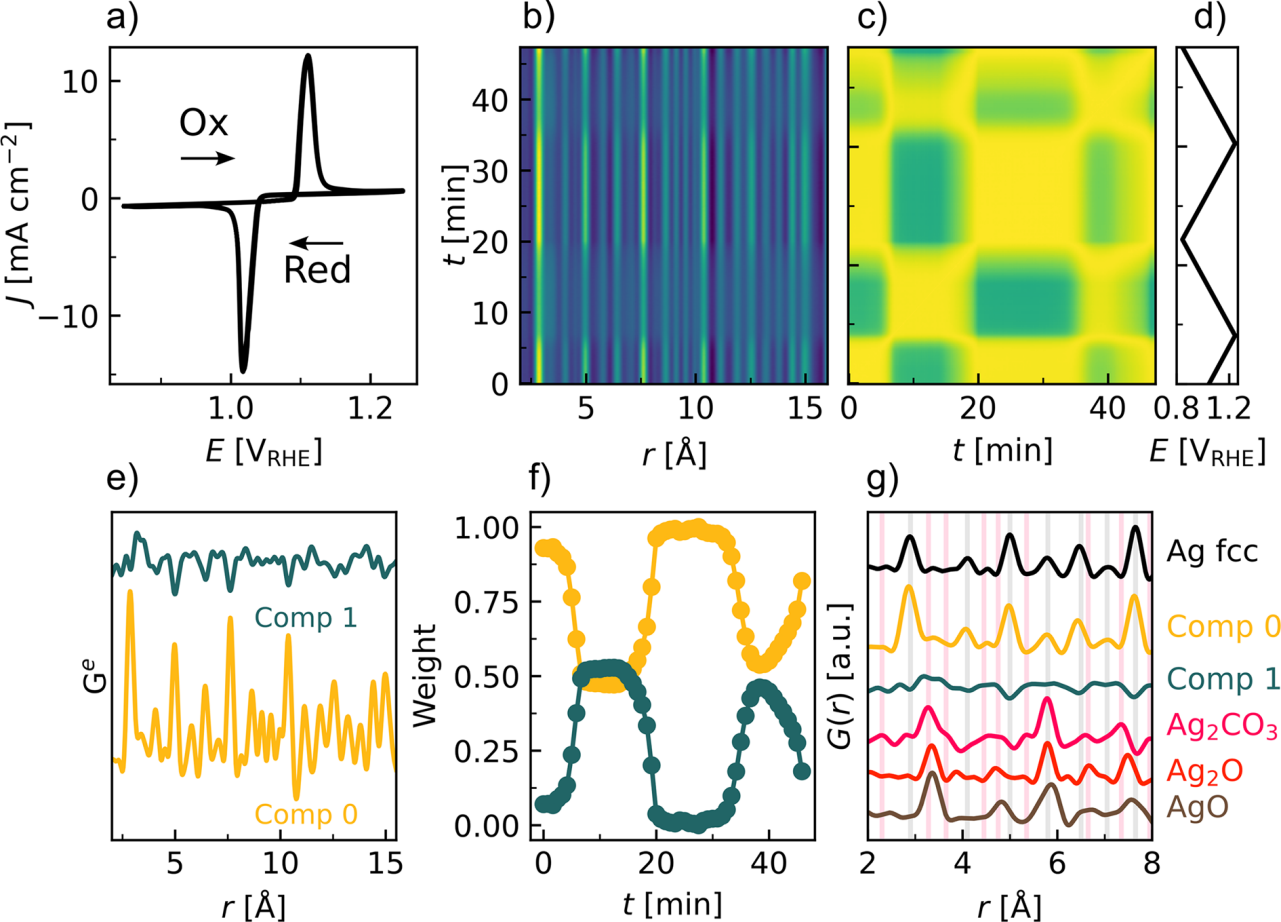
(*a*) CV performed with a scan rate of 0.5 mV s^−1^ in Ar-saturated electrolyte in a potential range starting in proximity to the open circuit potential, and in between 0.85 V_RHE_ and 1.25 V_RHE_. (*b*) Contour plot of the PDFs with a time resolution of 1 s. (*c*) PCC matrix between the time-resolved PDFs with an *r* range 0–100 Å, indicating structurally distinct regions. The color scale goes from blue, which represents the most dissimilar areas with a PCC of −0.95, to yellow corresponding to a PCC of 1. (*d*) Potential versus time plot indicating the time of the switching potentials. (*e*) Two NMF components from NMF analysis (Comp 0 and Comp 1) together with (*f*) the evolution of the components with time. (*g*) Comparison of the components with the calculated patterns of Ag f.c.c. [ICSD 64706 (Swanson & Tatge, 1953 ▸)], Ag_2_CO_3_ [ICSD 8011 (Masse *et al.*, 1979 ▸)], Ag_2_O [ICSD 20368 (Vereshchagin *et al.*, 1963 ▸)] and AgO [ICSD 27659 (McMillan, 1960 ▸)]. Vertical lines represent characteristic peaks for Ag f.c.c. (black) and Ag_2_CO_3_ (pink).

In the OER region, the catalyst was stepped to oxidative potentials in Ar atmosphere to investigate the redox process of Ag and its stability during operation in the anodic region. The current density and the total scattering function (Fig. S11) indicate gradual changes at higher potentials, with Bragg peaks from an additional phase appearing and an increase in the amorphous content at potentials above 0.9 V_RHE_. The structural changes in the PDF [Fig. 2 ▸(*d*)] are highlighted in gray, with an increase in correlations at 3.4, 4.5 and 7.0 Å, attributed to silver oxide or silver carbonate [Fig. 3 ▸(*g*)]. Hence, from a qualitative inspection of the PDFs, the applied anodic potential results in an oxidation of the Ag catalyst, apparent as significant changes to the local structure. In addition, the time evolution (Fig. S10) and NMF analysis (Fig. S12) indicate continuous structural changes at 1.7 V_RHE_, *i.e.* the structure has not reached a steady state at this potential.

The electrochemically active surface area and the double-layer capacitance have been determined from CV measurements in the non-faradaic region (Fig. S4 and Section S1.2, supporting information). From the measured double-layer capacitance of *C*_DL_ = 0.059 F, the number of atoms participating in the electrocatalytic surface reactions can be estimated as 4.5 × 10^16^ Ag atoms cm^−2^. Considering the geo­metrical dimensions of the X-ray beam (approximately 1 mm^2^), the number of electrochemically active Ag atoms probed by the X-rays is ∼4.5 × 10^14^ Ag atoms.

The transient behavior during the redox process was followed with CV with a scan rate of 0.5 mV s^−1^ and a time resolution of 1 s. The CV [Fig. 3 ▸(*a*)] exhibits sharp oxidation (anodic) and reduction (cathodic) peaks, with peak potentials at *E*_p,a_ = 1.1 V_RHE_ and *E*_p,c_ = 1.0 V_RHE_, respectively, and a peak separation of ∼92 mV indicating electrochemical (quasi-)reversibility. The peak shape hints at a quasi-reversible process with the sharp redox peaks followed by an extended tail (present at ∼80 mV above the peak potentials). Integrating the area under each peak results in a charge of 0.48 and 0.49 C cm^−2^, *i.e.* varying less than 2%. Thus, from the three fingerprints of full reversibility, the process is identified as at least quasi-reversible. From the integrated charge, and assuming the redox process is involving two electrons (Section S1.3, supporting information), the number of atoms active in the redox process is estimated as 1.5 × 10^18^ Ag atoms cm^−2^. As expected, this number is higher than the estimated number of Ag atoms participating in the electrocatalytic reactions at the surface. By again considering the geometrical dimensions of the X-ray beam, the number of redox-active Ag atoms probed by the X-ray is found to be ∼1.5 × 10^16^ Ag atoms.

The time evolution of the PDFs during CV [Fig. 3 ▸(*b*)] clearly shows changes to the local structure as the potential is cycled. PCC analysis is performed to identify the changes throughout the cycle [Fig. 3 ▸(*c*)]. The PCC (Myers *et al.*, 2010 ▸) is being used increasingly to study time-resolved PDFs, and the analysis can be performed on the *PDFitc* platform (Yang *et al.*, 2021 ▸). It is a measure of the linear correlations in data sets, *i.e.* the similarity between different frames, and is a useful method for studying correlations in large data sets (Kjaer *et al.*, 2022 ▸). The PCC takes a value between −1 and 1, corresponding to opposite behavior or a perfect linear correlation, respectively. Four regions are observed in the PCC matrix, which nicely fits with the scanned potential profile [Fig. 3 ▸(*d*)].

The correlation between frames within each redox region, *i.e.* above *E*_p,c_ and below *E*_p,a_, is almost unity, indicating the formation of a stable region. This suggests that no significant changes occur within each redox region. From the PCC two distinct stages of the redox reaction, which are cycled between, can be identified. The lowest PCC value is −0.95, indicating significant and anti-correlated differences between the two stages.

The structural evolution is further quantified with NMF analysis [Figs. 3 ▸(*e*) and 3 ▸(*f*)]. Different numbers of components are tested (Fig. S13). The simplest two-component analysis seems to sufficiently describe the changes [Fig. 3 ▸(*e*)], where one component (Comp 0) represents the reduced structure, and the other component (Comp 1) represents the structural changes at oxidizing potentials. The most prominent differences between the components are the appearance of a split peak at 3.4 Å in Comp 1 and inverse peaks exactly at the positions of the most intense peaks in Comp 0, indicating a decrease in their relative intensity with oxidation. The peak at 3.4 Å was also observed at *E* > 0.9 V_RHE_ during the OER experiment [Fig. 2 ▸(*d*)].

The evolution of components [Fig. 3 ▸(*f*)] shows how the structure is initially reduced as the weight fraction of Comp 0 is close to unity. As the potential approaches the anodic peak potential *E*_p,a_, the weight fraction of Comp 1 increases as a result of oxidation. At potentials *E* > *E*_p,a_ the weight fractions are constant at ∼0.5 for each component. Upon returning to a reducing potential, *E* < *E*_p,c_, Comp 1 again approaches unity. Such cyclical changes are observed within the entire potential range when cycling back and forth. As CV is a dynamic technique, the structure is not expected to return to its exact starting point; however, it is noteworthy that the cyclical structural changes can be directly correlated with the applied potential and the electrochemical redox processes.

The local structure of the components is compared with the calculated structure for different reference structures [Fig. 3 ▸(*g*)]. The local structure of Comp 0 is entirely described by Ag f.c.c. (face-centered cubic). The peak at 3.4 Å in Comp 1 can be described by the Ag–Ag correlation seen in AgO, Ag_2_O and Ag_2_CO_3_. In addition, the other peaks in Comp 1 can be described with either the oxide or carbonate structures, and it is difficult to make a certain distinction as to whether the oxidized state consists of one or a combination of the three phases. However, when combined with other analyses, such questions could be answered, as discussed in Sections 3.2 and 3.3.

Thus, with the AUREX cell, it is possible to measure total scattering data and perform PDF analysis to monitor the structure of the working catalyst. During the eCO_2_RR, structural changes were limited to a slight lattice contraction and particle growth, consistent with Ostwald ripening and coalescence occurring at the surface. By probing the bulk electrochemical response at the faradaic region, the quasi-reversible redox-reaction-induced phase transition could be followed.

### 3.2. Powder XRD

Operation at the cathodic potential during the eCO_2_RR resulted in few structural changes. Therefore, the focus in the following is on the steady-state behavior at the edges of the CV potential range, allowing the study of structural changes induced in the redox region. The structural changes at more extreme potentials are expected to be small in comparison. By following the behavior in the redox region, the oxidation mechanism is elucidated, as well as the formation mechanism and stability of the oxide/carbonate-derived Ag catalysts from a Ag f.c.c. pre-catalyst (Ma *et al.*, 2018 ▸).

XRD measurements were performed during 45 min of anodic potential followed by 15 min of a cathodic pulse. The anodic pulse potential was chosen to be sufficiently positive that Ag was oxidized (at 1.25 V_RHE_) and the cathodic pulse to be just below the reduction peak (at 0.85 V_RHE_). The experiments were performed with X-ray energies of both 60 keV at P02.1 at PETRA III, Germany, and 20 keV at DanMAX, MAX IV, Sweden.

#### 3.2.1. 60 keV

**Figure 4:**
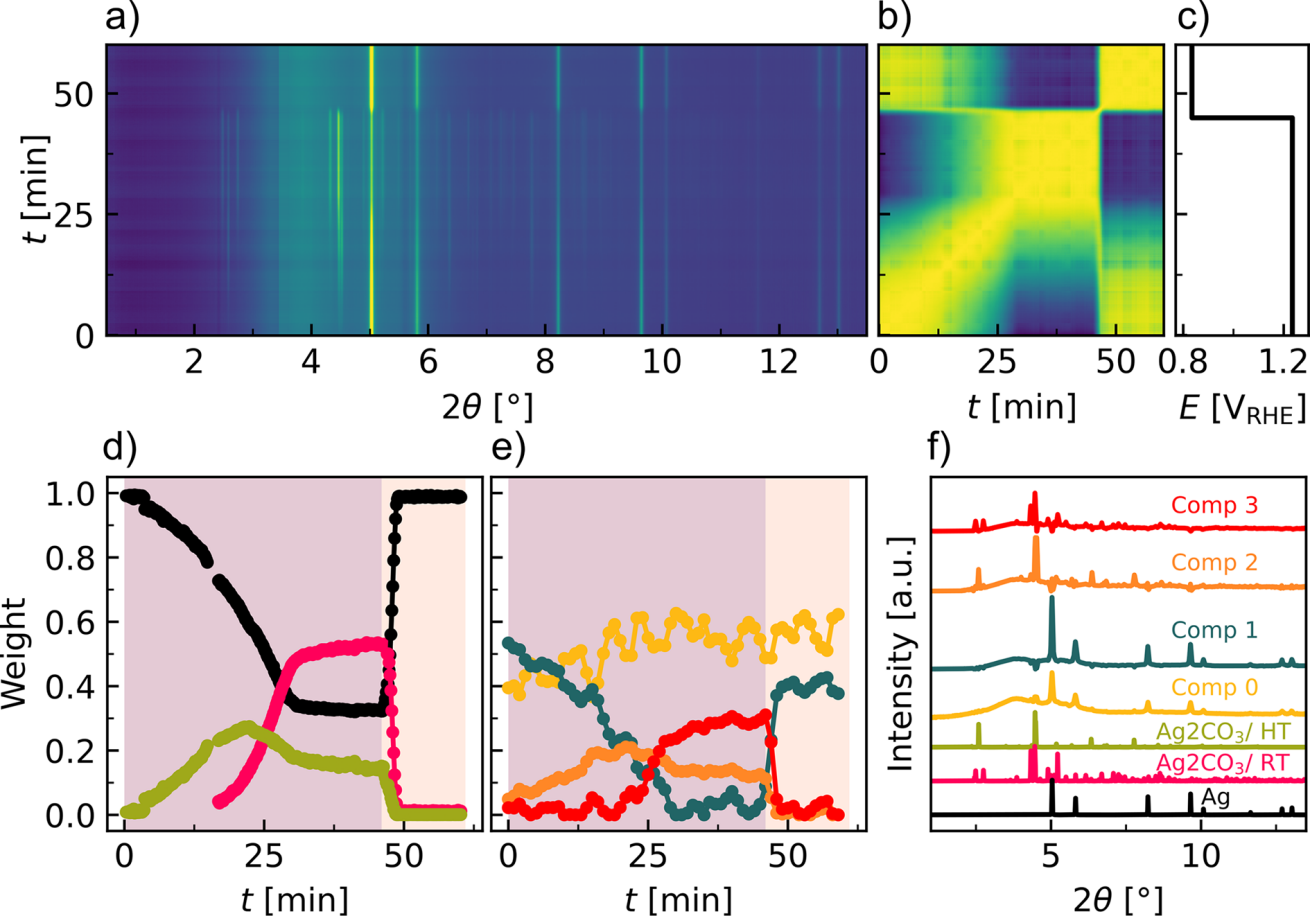
*Operando* XRD data (λ = 0.207 Å) measured during prolonged anodic etching (45 min at 1.25 V_RHE_) followed by reducing potentials (15 min at 0.85 V_RHE_). (*a*) Contour map of the time evolution of XRD data during the experiment. (*b*) PCC matrix between the time-resolved XRD data, indicating structurally distinct regions. The color scale goes from blue, which represents the most dissimilar areas with a PCC of 0.89, to yellow, corresponding to a PCC of 1. (*c*) Pulsed potential profile. (*d*) Weight fractions obtained from a three-phase sequential Rietveld refinement against Ag f.c.c. in black [ICSD 64706 (Swanson & Tatge, 1953 ▸)], Ag_2_CO_3_/HT in green [ICSD 281043 (Norby *et al.*, 2002 ▸)] and Ag_2_CO_3_/RT in pink [ICSD 8011 (Masse *et al.*, 1979 ▸)]. The lavender and pale-orange background colors represent the regions biased at potentials of 1.25 and 0.85 V_RHE_, respectively. The error bars on the weight fractions are smaller than the width of the markers and therefore have not been included in this plot. (*e*) NMF mapping with four components performed on the same data. (*f*) Calculated diffraction patterns of the Ag f.c.c. phase, the two Ag_2_CO_3_ phases and the four NMF components determined from NMF mapping.

The time evolution of the XRD patterns is shown in the contour map in Fig. 4 ▸(*a*), along with the PCC matrix [Fig. 4 ▸(*b*)]. The oxidation process is gradual until ∼27 min, where a stable composition has been achieved. Upon entering the cathodic region, the material quickly reverts to its original structure. A magnification of the low-angle region (Fig. S14) indicates the immediate formation of a new Bragg peak at 2.7°, followed by two additional peaks at 2.5° and 2.8° emerging after ∼18 min at the anodic potential of 1.25 V_RHE_. The two different time constants for the peak formation indicate the formation of two different phases. The two new phases are expected to form due to the oxidizing conditions and interaction with the KHCO_3_/K_2_CO_3_ electrolyte. Understanding the formation and kinetics of these phases can help in elucidating the degradation/dissolution mechanism of Ag, and hence its stability under working conditions.

A fingerprinting analysis of the most likely Ag oxides and carbonates reveals that all three low-angle peaks can be described by two different Ag_2_CO_3_ phases (Figs. S14 and S15, Table S2): a monoclinic phase (space group *P*12_1_/*m*1, No. 11), which is the most common silver carbonate (Masse *et al.*, 1979 ▸), as well as a trigonal carbonate phase (space group *P*31*c*, No. 159), which was originally published as a high-temperature phase (Norby *et al.*, 2002 ▸). The monoclinic phase is referred to as Ag_2_CO_3_/RT (room temperature) and the trigonal phase as Ag_2_CO_3_/HT (high temperature) herein. To our knowledge, the Ag_2_CO_3_/RT phase has not been reported under *operando/in situ* electrocatalytic conditions before. However, previous *ex situ* studies of Ag nanoparticle catalysts have shown the presence of the Ag_2_CO_3_/RT phase after an anodic etching process. Notably, this phase was responsible for lowering the overpotential for the eCO_2_RR, thus reducing the required energy input for the reaction (Ma *et al.*, 2018 ▸). On the other hand, the formation of the Ag_2_CO_3_/HT phase has not been previously documented under redox conditions. Therefore, the influence of this additional Ag_2_CO_3_/HT phase on the overpotentials for both eCO_2_RR and OER has yet to be systematically explored. Furthermore, a comparative assessment of the electrochemical performances of Ag-derived Ag_2_CO_3_ catalysts relative to as-prepared Ag_2_CO_3_ catalysts is needed. It is anticipated that these phases form at potentials more positive than the anodic peak potential, *E*_p,a_. A higher positive potential is expected to increase the reaction kinetics and accelerate the formation process.

A three-phase sequential refinement was performed using the two Ag_2_CO_3_ phases and the original Ag f.c.c. structure to elucidate the formation mechanism of the carbonates (Fig. S16). Details on the refinement parameters and procedure are presented in the supporting information (Section S3.1.1). The evolution of the refined weight fractions clearly shows the formation of the Ag_2_CO_3_/HT phase already after 2–3 min, long before the Ag_2_CO_3_/RT phase starts to form at ∼17 min [Fig. 4 ▸(*d*)]. After the formation of the Ag_2_CO_3_/RT phase, the fraction of the Ag_2_CO_3_/HT phase decreases. A gradual drop in the fraction of Ag f.c.c. occurs as soon as the experiment is started, until ∼27 min where it stabilizes with a weight per cent of 38%, along with a stabilization of the carbonate weight fractions, indicating a steady state is reached. At 45 min, when the potential is switched from oxidative to reductive, an immediate decrease of the carbonate phases is observed, with the weight fractions approaching 0% within 3 min. Thus, the structural transformations during the reduction process are significantly faster than those during the oxidation process.

The lattice parameters for the Ag f.c.c. and Ag_2_CO_3_/RT phases are relatively constant during the entire oxidative potential (Fig. S17). However, a minor increase in the unit-cell parameter of the Ag phase is observed during the oxidation, followed by a decrease upon reduction, although the total change during the experiment is notably small (on the fourth decimal). If the unit-cell change had been due to thermal lattice expansion alone, it would only account for a temperature change of around 3 K (Bogatyrenko & Kryshtal, 2021 ▸). Lattice expansion and strain have been attributed to the adsorption of electroactive species (Mistry *et al.*, 2016 ▸). The adsorption of carbonate anions on the Ag catalyst surface is expected to be a first step in the Ag_2_CO_3_ formation mechanism.

For the Ag_2_CO_3_/HT phase, the unit cell expands slightly along *a* and contracts along *c* during oxidative potential, indicative of an anisotropic lattice distortion.

The *R*_Bragg_ parameter signifies the goodness of fit for each of the phases and shows good fits for the Ag phase over the entire time duration of the experiment, while the carbonate phases need to be present at sufficiently high weight fractions before reliable fits are obtained.

For comparison, NMF analysis is performed with two to four components included [Fig. 4 ▸(*e*) and Fig. S18]. The reconstruction error indicates that just two components describe the system well; however, by including three or four components a slight improvement is observed. Further, with four components the shape of the evolution of weight fractions from the sequential refinement can be reproduced with a striking similarity.

Comp 0 and 1 represent the initial structure and share all the Bragg peaks from the Ag f.c.c. structure, with slightly different backgrounds and anti-correlated intensities at ∼4.8° [Fig. 4 ▸(*f*)]. The weight of Comp 0 is close to constant throughout the experiment, with small oscillations, whereas Comp 1 decreases in weight fraction as expected for a component describing the Ag f.c.c. structure.

Comp 2 shares a high likeness with the trigonal Ag_2_CO_3_/HT phase, noticeable from the distinct low-angle peak at 2.7°. The formation of this component is also initiated within the first few minutes of the experiments and drops off slightly as Comp 3 starts to increase at ∼20 min. Comp 3 describes the monoclinic Ag_2_CO_3_/RT phase. Where the sequential refinement resulted in a weight per cent of ∼50% of this phase, Comp 3 from the NMF analysis only reaches ∼30%. Thus, NMF mapping will not give the absolute sample composition, and in general the components are not real physical phases, as is the case here with the presence of negative peak intensities relative to the background. However, it provides a reliable picture of the mechanisms at play. Given the ease of the analysis compared with the sequential Rietveld refinement, it can be a valuable initial analysis to gather information about the time evolution of the system. Furthermore, it is a strong tool for data analysis if compounds with structures with no database match are formed.

The structural evolution of both the refined weight fractions and the NMF components is compatible with a system of pseudo-first-order kinetics, where the f.c.c. fraction reacts to form the two carbonates independently coupled with an equilibrium reaction between the RT and HT phases, which is heavily shifted towards the RT phase.

The morphology of the electrode was investigated with scanning electron microscopy before and after the prolonged anodic etching, 45 min at 1.25 V_RHE_ followed by the cathodic pulse at 0.85 V_RHE_ for 15 min (Fig. S23). No significant changes are observed and, despite a few cracks in the surface structure, the microstructure appears preserved. This is in contrast to previous results on nanostructuring of the surface of Ag foils with anodic etching; however, these experiments were performed at more extreme anodic potentials, and on metal foils (Ma *et al.*, 2016 ▸, 2018 ▸). As the Ag catalyst used in this experiment series is initially nano­structured, a marked difference between the before and after is not expected (Ma *et al.*, 2016 ▸, 2018 ▸). From energy-dispersive X-ray spectroscopy analysis, a decrease of the Ag atomic per cent of 3% during the experiment is found (Table S3).

The dissolution during the anodic potential was investigated with ICP–OES, and the concentration of Ag in the electrolyte from the working electrode side is determined to be 0.0883 (5) mg l^−1^ (0.6% of the original Ag loading) with no other elemental traces. This implies a partial catalyst dissolution and/or erosion, indicating that the species formed under the anodic potential are unstable (Timoshenko *et al.*, 2022 ▸; Schalenbach *et al.*, 2018 ▸). Comparing the dissolution with the solubility product of Ag_2_CO_3_ of *K*_sp_ = 8.46 × 10^−12^, it is expected that when switching to the cathodic potential a partial stripping of the passivating carbonate layer occurs.

Thus, with the AUREX cell, it is possible to measure XRD data with a high signal-to-noise ratio, ensured by the minimal background contributions from the cell. During the prolonged anodic etching experiment, the formation of two Ag_2_CO_3_ phases could be followed. The evolution of these could be tracked with sequential refinements and NMF analysis, both of which suggest a system of pseudo-first-order kinetics. The switch to a cathodic potential was found to immediately revert the structure to Ag f.c.c., associated with a partial catalyst dissolution and/or erosion.

#### 3.2.2. Beam-induced effects at 20 keV

Beam-induced effects (also known as beam damage) are a well known phenomenon for *operando* battery experiments, where the high energy and flux of the synchrotron X-ray radiation may interfere with the components of the cell and hinder the electrochemical reaction of interest, by *e.g.* creating photoelectrons which can have a reducing effect (Borkiewicz *et al.*, 2015 ▸; Christensen *et al.*, 2023 ▸). The effect should be monitored before initiating an electrochemical experiment and can be established by exposing the catalyst system to the beam and observing whether changes occur to the scattered intensity where the system should be stable (Magnussen *et al.*, 2024 ▸). At 60 keV, this was not observed; however, at a lower X-ray energy of 20 keV, this was found to be a pronounced issue. This is attributed to an increase in the interaction cross section of the beam with the sample, which for Ag is approximately three times higher at 20 keV compared with 60 keV. The influence of the interaction between beam and sample on the redox reaction of the Ag catalyst was explored by performing an identical anodic etching experiment with a beam energy of 20 keV (Fig. S19).

The expected oxidation of the Ag f.c.c. phase to the two carbonate phases is not observed within the anticipated time frame, and only upon moving the position of the beam to an unexposed spot on the sample is the phase transition observed. In contrast, as the cathodic pulse is applied, the reduction process can be captured without moving the position. Thus, the reducing beam is hindering the oxidation process when we move against it by applying an anodic potential.

The reducing effect of the beam can be lowered by decreasing the overall dose by limiting the exposure time at each position. A pulsed potential experiment (switching between cathodic and anodic potentials every 5 min, for nine pulses) was performed as an accelerated stress test while the position was changed to a fresh spot every 60 s (Fig. S20). With an exposure time of 0.5 s followed by 2.5 s of wait time for each frame, the resulting time resolution was 3 s, and each position was exposed to the 20 keV beam for a total time of 10 s.

A clear difference is observed when the potential is working with the beam [cathodic, Fig. S20(*e*)] versus against the beam [anodic, Fig. S20(*d*)]. The oxidation is only visible when the position is moved to a fresh spot, while the reduction is visible in between position changes. This is further quantified with NMF analysis (Figs. S21 and S22). Periodic reversible changes in the catalyst structure and composition are shown, and the Ag_2_CO_3_ concentration is not accompanied by an accumulation of carbonate species. All carbonate species that are generated during the anodic potential pulse are removed during the subsequent cathodic pulse. This removal of electrochemically grown carbonate species appears fast. The reduction process is expected to be faster than the oxidation, and the reduction is achieved within 10–20 s, in contrast to a minute for oxidation; however, the exposure of merely 10 s at this energy still hinders the oxidation as a fresh sample position is more oxidized.

Using a lower X-ray energy for diffraction has the clear general advantage of enabling XRD data of decent quality for lighter elements. To take advantage of this, together with the possibility of higher time resolution at a beamline with higher flux and brilliance, position change after each exposure is a possible solution to the problem of beam-induced effects.

### 3.3. XAS

XAS provides element-specific information: the X-ray absorption near-edge structure (XANES) region of the spectra provides information on the oxidation states, while the extended X-ray fine structure (EXAFS) region can reveal the local structure around the absorbing atom. Combining this with simultaneous XRD measurements allows for short- and long-range-order information. *Operando* transmission XAS on the Ag *K* edge, *i.e.* at 25514 eV, was performed at Balder, MAX IV, Sweden, in sequential repeats with XRD just below the absorption edge at an X-ray energy of 25201 eV. XANES and EXAFS data up to *k* = 15 Å^−1^ were collected within a scan time of just 3 s followed by a 1 s XRD exposure, resulting in a total combined time resolution of 4 s. Details of the Balder beamline experimental setup for simultaneously combined XAS–XRD will be published separately.

**Figure 5:**
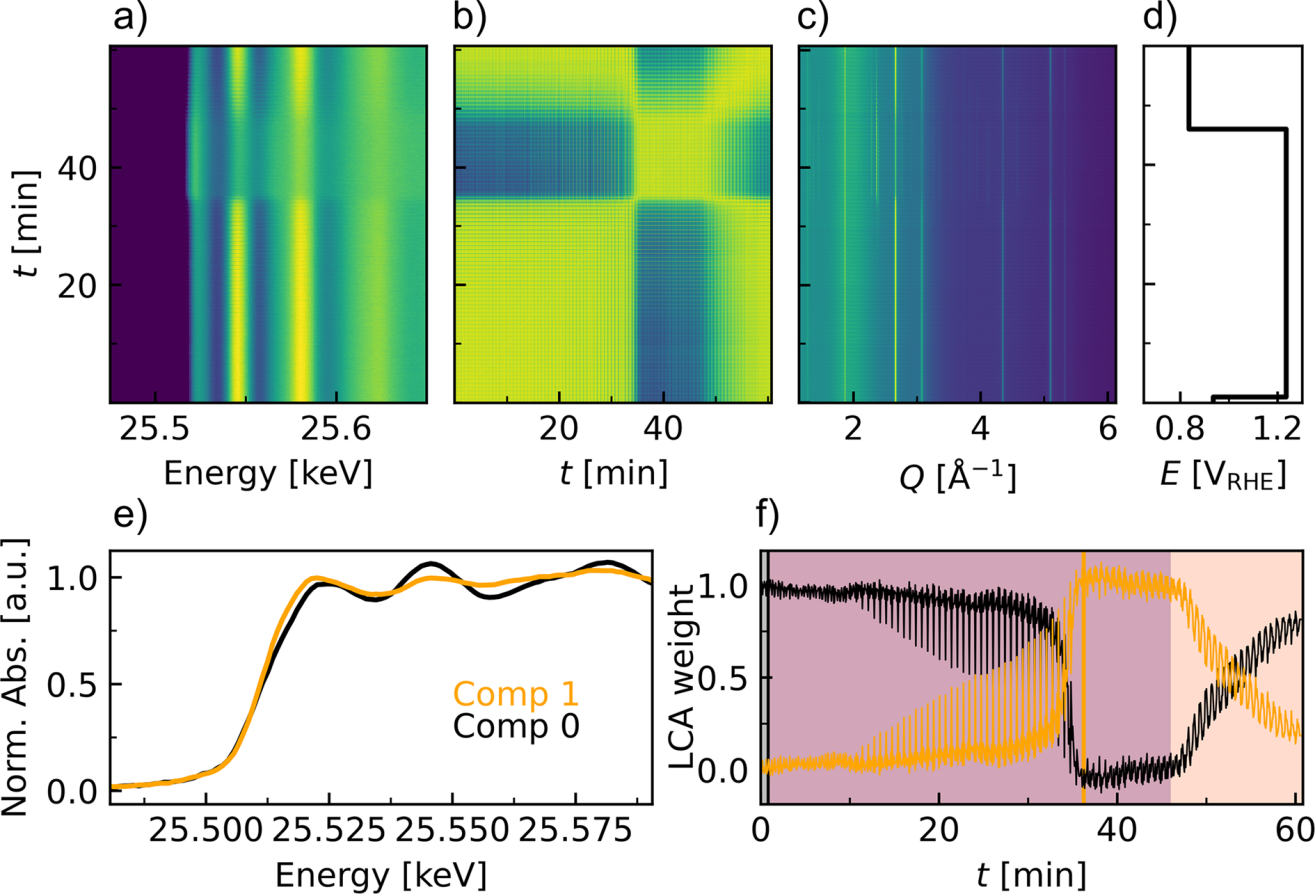
Multimodal XAS–XRD experiment performed under the *operando* conditions of prolonged anodic etching (45 min at 1.25 V_RHE_) and a subsequent cathodic pulse (15 min at 0.85 V_RHE_). Ten sample positions were probed repeatably. (*a*) Contour map over the time evolution of the normalized XANES data. (*b*) PCC matrix between the time-resolved XAS data, indicating structurally distinct regions. The color scale goes from blue, which represents the most dissimilar areas with a PCC of 0.999, to yellow, corresponding to a PCC of 1. (*c*) Time evolution of the XRD data recorded simultaneously with the XAS. (*d*) Pulsed potential profile. (*e*) Two internal reference spectra recorded during the *operando* experiment, used in the LCA. Comp 0 (black) is the starting structure, while Comp 1 (orange) is the most oxidized spectrum. (*f*) Evolution of the LCA components. The vertical black line (at 1 min) represents the position of Comp 0 in the data series, while the vertical orange line (at 37 min) shows the position of Comp 1. The shaded lavender region indicates the region where the anodic potential of 1.25 V_RHE_ is applied, and the pale-orange region indicates the change to the cathodic potential of 0.85 V_RHE_.

The anodic etching experiment, similar to that described for XRD, was performed while following the time evolution with the multimodal XAS–XRD (Fig. 5 ▸). Ten different sample positions were probed repeatedly with a beam of 100 µm in diameter to lower the X-ray dose at each spot and gain information about the statistical spread in position (Fig. S25). A contour map over the time evolution of the normalized XANES data [Fig. 5 ▸(*a*)] and the PCC matrix [Fig. 5 ▸(*b*)] show little change to the oxidation state until ∼36 min at 1.25 V_RHE_ potential hold, where the high energy of the Ag *K* edge results in a significant core-hole lifetime broadening, compared with 3*d* metal *K* edges, which reduces the energy resolution of the XANES. Thus, only a small but still significant variation is observed between oxidation states (Fig. S24). The high similarity between spectra throughout the *operando* experiment is further reflected in the lowest PCC value of 0.999, indicating that all spectra are quite similar.

A parallel evolution of additional Bragg peaks is observed with XRD. These new peaks can be assigned to the two Ag_2_CO_3_ phases. The XRD data similarly indicate a later onset of the carbonate formation at the anodic potential of ∼25 min at 1.25 V_RHE_ relative to the XRD experiment at 60 keV. Thus, there is an agreement between the structural description obtained on the local (XAS) and the long-range order (XRD) during the multimodal XAS–XRD experiment. However, the carbonate formation detected during the *operando* XRD measurement performed at 60 keV was quicker, as it occurred within the first 15 min of the anodic potential. In addition to the X-ray energy difference, a significant contrast between the two *operando* experiments is the size of the beam. The beam during the 60 keV measurement was mm-sized, while it was µm-sized for the XAS–XRD measurement. Thus, the data from the 60 keV experiments are averaged over a larger sample size than the XAS–XRD data.

LCA was performed against both internal reference spectra, from the reaction itself [Fig. 5 ▸(*e*)] and standard reference spectra (Fig. S26). The LCA against both internal and standard reference spectra gives similar information to the NMF analysis performed on the scattering data. It indicates gradual changes to the catalysts starting at approximately 10 min. A spread in behavior across the positions is observed, as some positions appear to oxidize quicker, which supports the hypothesis that part of the difference in carbonate formation times observed across beamlines can be explained by the footprint of the beam on the sample. Another factor is the small variation between samples, *e.g.* the exact amount of catalyst deposited, the homogeneity of the catalyst *etc.* As was seen from the XRD experiment at 60 keV, a steady state is reached, where the catalyst is not oxidized further. Upon applying the cathodic potential, the same positional spread is not observed during the reduction. The LCA fitting against the reference of Ag and Ag_2_CO_3_ indicates that ∼44% of the structure is left in the original Ag state (Figs. S26 and S27).

**Figure 6:**
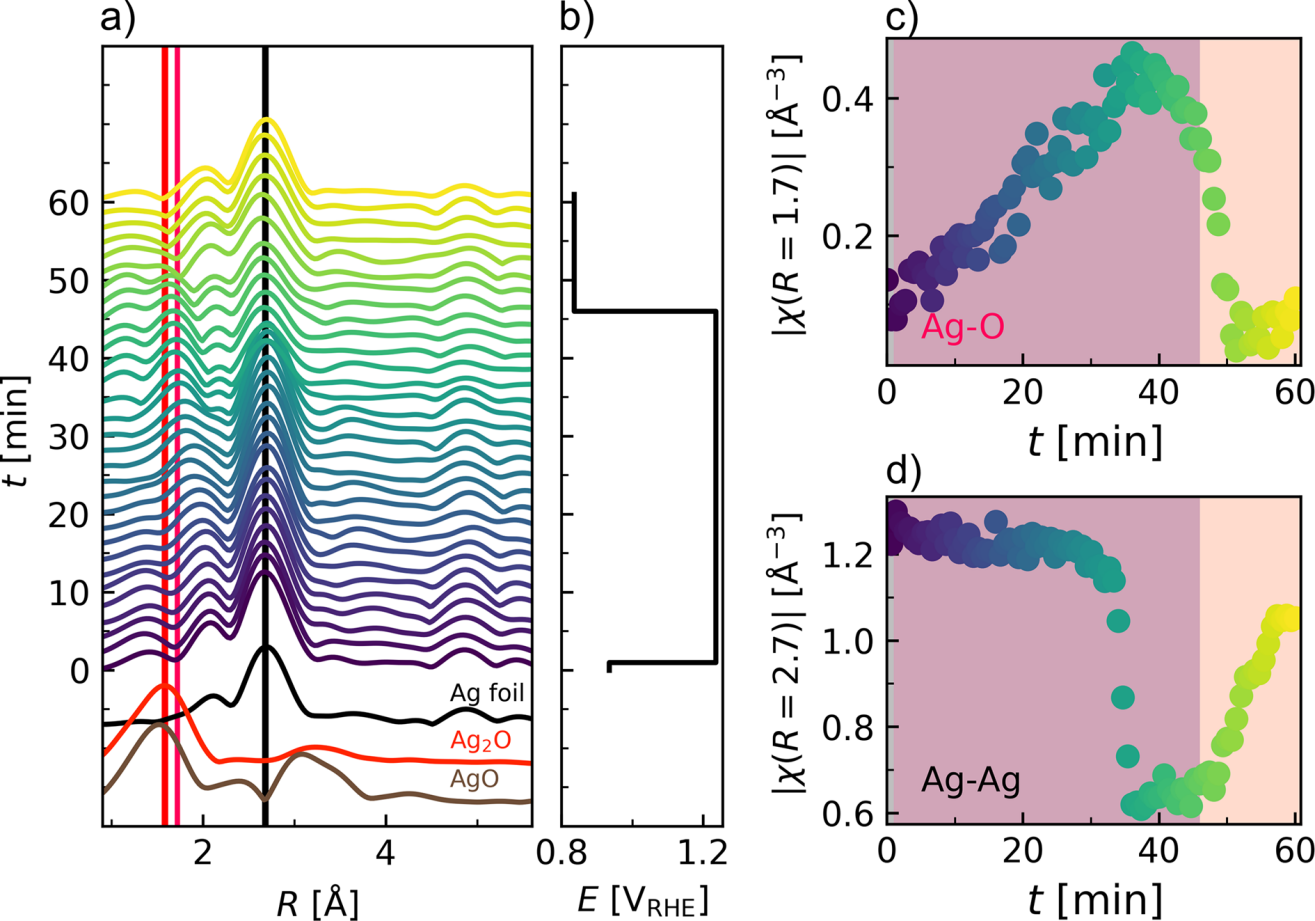
(*a*) Waterfall plot over the *R* space EXAFS region (not phase corrected) compared with reference spectra from a Ag foil, Ag_2_O and AgO. The vertical black line shows the position of the Ag–Ag back-scattering paths while the red and pink lines indicate the position of Ag–O as expected from Ag_2_O and Ag_2_CO_3_. (*b*) The pulsed potential profile. (*c*), (*d*) The evolution of the Fourier transform magnitude at the Ag–Ag and Ag–O back-scattering signal, respectively.

The EXAFS region is shown in *k* space (Fig. S28) and Fourier transformed to *R* space (Fig. 6 ▸, and Figs. S29 and S30). A good data quality is observed up to *k* = 15 Å^−1^.

The local structure around the absorbing Ag atom is elucidated and compared with the reference spectra in Fig. 6 ▸. The formation of a nearest-neighbor Ag—O bond is evident, while the Ag–Ag correlation is simultaneously decreasing. It can be observed that the maximum in the Fourier transform attributed to Ag–O interaction is at higher distances com­pared with those of Ag_2_O and AgO, indicative of a weaker binding as expected for Ag_2_CO_3_. Following the intensity of the Ag–Ag peaks gives a similar time evolution to the LCA fitting [Fig. 6 ▸(*d*)]. The intensity of the nearest-neighbor Ag–O peak in turn increases as soon as the oxidative potential is applied, indicating immediate changes to the local structure.

The local structural information obtained from XAS is complementary to that from the PDF. As the XAS experiment is only probing the correlations around Ag, it gives a much clearer picture of the change in the nearest-neighbor correlations. However, already past the second shell, *e.g.* the Ag–Ag correlation expected in Ag_2_CO_3_, the XAS signal is much less sensitive. At the same time, the nearest-neighbor Ag–O correlation is more difficult to resolve with PDF analysis, due to the lower scattering power of oxygen. Thus, the utilization of both techniques in combination, as is possible with the AUREX *operando* cell, provides a more coherent description and a better understanding of the structural transformations, while the long-range order is best probed with XRD.

## 4. Conclusion

The AUREX *operando* electrochemical flow cell has been developed and serves as a versatile setup in characterizing the active phase of electrocatalysts under the working conditions of an applied potential, contact with the liquid electrolyte and local reaction environment. The low background contributions provide excellent signal-to-noise ratios, and the uniform flow field ensures a continuous supply of fresh electrolyte (including CO_2_ for eCO_2_RR studies) in addition to the removal of gas bubbles formed during experiments. The cell is easy to use and the size of the X-ray window allows for multiple sample positions to be probed, thereby lowering the beam exposure, which is important to minimize beam-induced effects. The use of the cell has been demonstrated with total scattering and PDF analysis, XRD, and multimodal XAS–XRD.

With the combination of these characterization tools, it is possible to develop a comprehensive understanding of reaction processes within electrode materials for electrocatalysis, including the short-range ordering and characterization of amorphous and crystalline intermediate phases. It is demonstrated that the data obtained with the *operando* cell are suited for both direct-space PDF refinements and reciprocal-space Rietveld refinements, with a time resolution of seconds.

The *operando* experiments performed on the commercial Ag catalyst have elucidated its active structure during the eCO_2_RR where Ag is stable and exhibits a small increase in particle size, in accordance with previous studies. The structural changes and limited stability under anodic conditions have been identified. The reversible redox processes have been followed during CV, and the formation of two different Ag_2_CO_3_ phases with different formation times and mechanisms has been revealed upon holding an oxidative potential just above the anodic peak potential. To the best of our knowledge, neither of these two phases has been identified under electrocatalytic *operando* conditions before. The bulk structural changes can be followed by focusing on the redox region, while the surface structural changes at the more extreme working potentials of electrocatalysis are limited to changes in lattice parameters (*i.e.* bond lengths), particle size and disorder. Detailed knowledge of the structural changes, lattice contraction and phase transformations of electrocatalysts under commercially relevant electrical potential biases is critical for the rational design of novel and improved materials. This understanding helps maximize both material activity and utilization, advancing the development of new energy conversion technologies.

This work serves as a proof of concept of the strength of combining multiple synchrotron X-ray techniques for electrochemical *operando* studies to gain an understanding of the local-, medium- and long-range order in the catalyst under working conditions, as is achievable with the AUREX cell, in addition to exemplifying the valuable insights from both model-free and model-based analysis techniques.

## 5. Related literature

The following references are cited in the supporting information for this article: Beesk *et al.* (1981 ▸), Harris *et al.* (2020 ▸), Ishii *et al.* (2021 ▸, 2023 ▸), Jansen & Fischer (1988 ▸), Juhás *et al.* (2013 ▸, 2015 ▸), Kieffer *et al.* (2020 ▸), Klementiev *et al.* (2016 ▸), Macrae *et al.* (2020 ▸), Newville (2013 ▸), Niggli (1922 ▸), Rodríguez-Carvajal (1993 ▸), Sagadevan *et al.* (2023 ▸), Salkind & Zeek (1959 ▸), Schökel *et al.* (2021 ▸), Standke & Jansen (1986 ▸, 1987 ▸), Stehlík & Weidenthaler (1959 ▸), Stehlík *et al.* (1959 ▸), Suzuki (1960 ▸), Thatcher *et al.* (2022 ▸), Wyckoff (1922 ▸), Yang *et al.* (2014 ▸) and Yoon *et al.* (2018 ▸).

## Supplementary Material

Supporting information. DOI: 10.1107/S1600576724007817/oc5036sup1.pdf
