# Supplementary material for: The AUREX cell: a versatile operando electrochemical cell for studying catalytic materials using X-ray diffraction, total scattering and X-ray absorption spectroscopy under working conditions
Source: J Appl Crystallogr. 2024 Sep 20;57(Pt 5):1489–502. doi: 10.1107/S1600576724007817 (PMC11460379; doi:10.1107/S1600576724007817)
Supplement: Supplementary file 1 [file j-57-01489-sup1.pdf]

JOURNAL OF
APPLIED
CRYSTALLOGRAPHY

Volume 57 (2024)

Supporting information for article:

The AUREX cell: a versatile *operando* electrochemical cell for studying catalytic materials using X-ray diffraction, total scattering and X-ray absorption spectroscopy under working conditions

Sara Frank, Marcel Ceccato, Henrik S. Jeppesen, Melissa J. Marks, Mads L. N. Nielsen, Ronghui Lu, Jens Jakob Gammelgaard, Jonathan Quinson, Ruchi Sharma, Julie S. Jensen, Sara Hjelme, Cecilie Friberg Klysner, Simon J. L. Billinge, Justus Just, Frederik H. Gjørup, Jacopo Catalano and Nina Lock

S1. Electrochemistry

All electrochemistry experiments were performed with a CHI660E electrochemical workstation in the three-electrode *operando* flow cell.

All experiments were performed in 0.1 M $KHCO_3$ and 0.1 M K_2CO_3 (both compounds were bought from Sigma Aldrich without further purification) electrolyte buffer solution, saturated with either Ar 5.0 (High Purity ≥ 99.999%) (pH = 9.3) or CO_2 (Super-critical Fluid grade, Purity ≥ 99.998%). The electrolyte was pumped to each half-cell from two separate reservoirs with a volume of 500 mL, constantly bubbled with either Ar or CO_2.

The potential recorded with respect to Ag/AgCl was converted *versus* the reversible hydrogen electrode (RHE) through the following equation:

$$E(\mathrm{V\ vs\ RHE}) = E^0(\mathrm{V\ vs\ NHE}) + E^0(\mathrm{V\ vs\ Ag/AgCl}) \ + 0.059 \times \ \mathrm{pH}$$

where $E^0(\mathrm{V\ vs\ NHE}) = 0.198\ \mathrm{V}$.

S1.1. The ohmic and internal resistance of the cell

The cell resistance is measured with electrochemical impedance spectroscopy (EIS) at a potential close to the open circuit potential (OCP) of ~ 350 mV, in Ar and CO_2 saturated atmosphere, both with and without flow.

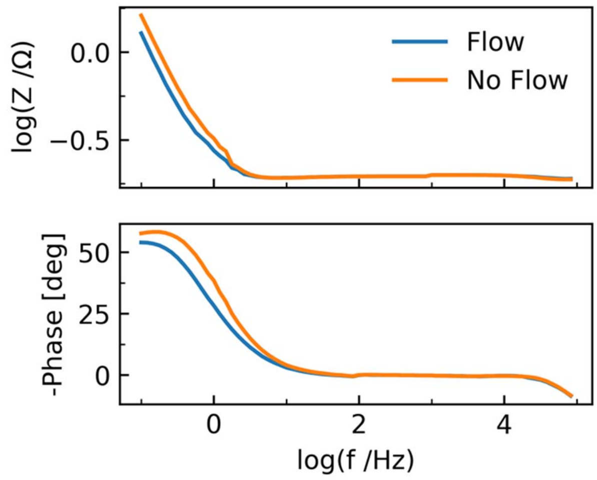

Figure S1 Bode plot recorded with the cell in flow operation and without flow in Ar atmosphere

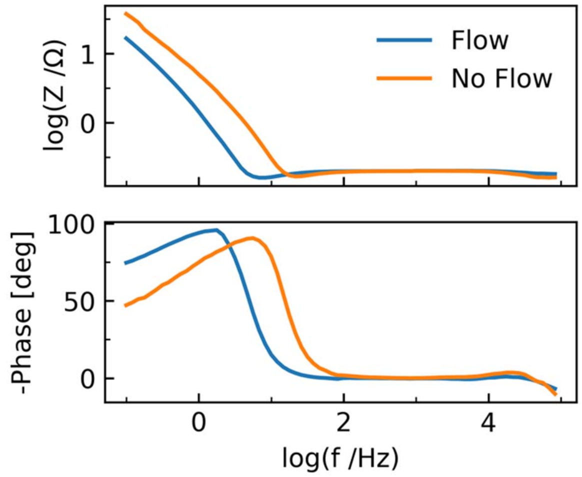

Figure S2 Bode plot recorded with the cell in flow operation and without flow in CO_2 atmosphere.

The ohmic resistance can be measured from the Z_{re} at high frequency ($f \in [10^2; 10^4]$ Hz) when the phase is $\varphi \sim 0$. This resistance includes all the electronic and ionic contributions of the cables, current collectors, terminals, and electrodes (including the contact resistances between these components) and electrolyte and membrane. From Figure S1 and S2 we measured a value of $R_s =$

$0.197(1)\ \Omega$. Afterwards, at lower frequencies ($f \in [10; 10^2]$ Hz), the contributions from the charge transfer resistances at the anode and cathode can be seen. The charge transfer resistance (anode plus cathode) was estimated to be $0.07\ \Omega$ by fitting the data by using a Randles equivalent electrical circuit with a constant phase element (see Figure S3). Therefore the (total) internal resistance of the *operando* cell was measured to be $R_{\text{in}} = 0.27\ \Omega$. This value compares quite well with the one measured during the *operando* experiments at the synchrotron facilities from iR compensation tests ($R_{\text{in}} = 0.4 - 0.6\ \Omega$). Lastly, at frequencies $f < 1$ Hz the system starts to be mass transfer-limited as the Nyquist plot becomes a straight line.

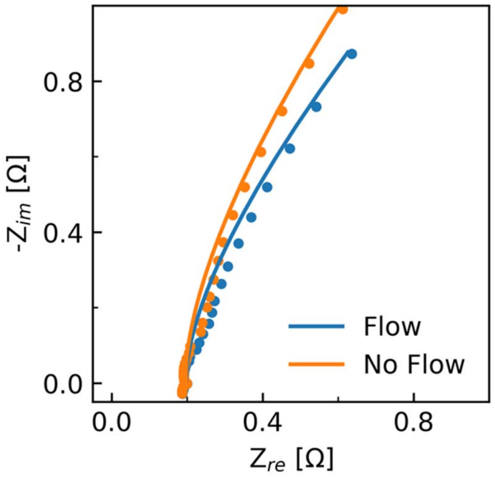

Figure S3 Nyquist plot over the impedance measured in Ar saturated electrolyte, with and without flow.

S1.2. The double layer capacitance

The double layer capacitance C_{DL} is estimated from CVs recorded in a non-faradaic region ($E = 0.6 \pm 0.1\ V$) with different scan rates in CO_2 saturated 0.1 M $KHCO_3$/ K_2CO_3 electrolyte. The C_{DL} is determined as the average of the slope from a linear fit of the scan rate *vs* the current density to both the cathodic and anodic sweeps. From Figure S4, this is determined as:

$C_{DL} = 0.059$ F.

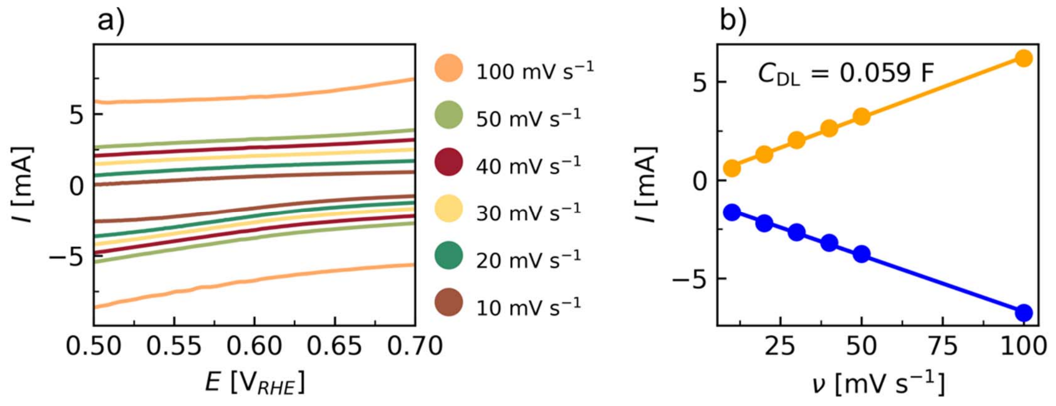

Figure S4 Estimation of the double layer capacitance. a) CVs recorded with different scan rates. b) The current density recorded at $E = 0.6$ V_{RHE} *vs* the scan rate.

The electrochemically active surface area (*ECSA*) is determined from the equation:

$$ECSA = C_{DL}/C_S$$

Where C_S is the specific capacitance of the sample. This value is taken as 40 μF cm^{-2} which is the accepted value for Ag in an aqueous electrolyte (Yoon *et al.*, 2018). With these values the $ECSA \sim 1478\ cm^2$ (or $300\ cm^2/mg$) which is higher but of the same order of magnitude reported in the literature for this catalyst (Kutz *et al.*, 2017). Following the ICP-OES measurements the number of Ag atoms per geometrical cm^2 was estimated to 7.8×10^{18}.

With a beam size of 1×1 mm^2 (60 keV at P02.1, DESY), the number of Ag atoms probed by the X-ray beam is estimated as 7.8×10^{16} Ag atoms. With a beam size of 500 μm (FWHM), (20 keV at DanMAX, MAX IV), 1.85×10^{16} Ag atoms are probed, and a beam size of 100×100 μm^2 (25.514 keV at Balder, MAX IV) will probe 7.8×10^{14} Ag atoms.

S1.3. Cyclic Voltammetry

The charge Q, involved in a redox process can be determined by integrating the area under the oxidation/ reduction peak, according to the below equation:

$$Q = \int i\,dt = \int i\,dv \frac{dt}{dv} = \frac{1}{v}\int i\,dv$$

Where i is the current and v is the scan rate.

The number of atoms participating in the redox reaction, N, is determined as:

$$N = \frac{Q}{e\,n}$$

Where $e = 1.602176634 \times 10^{-19}$ C, is the elemental charge of 1 electron and n is the number of electrons involved in the redox process.

The number of electrons involved in the redox process, n, is estimated from CV recorded with a faster scan rate of 20 mV s^{-1} (Figure S5a). A scan rate higher than the one used in the *operando* experiments (and reported in Figure 1c of the manuscript) was needed to increase resolution and better resolve the redox peak. From Figure S5a, two distinguishable peaks are clearly visible; therefore, it is assumed that $n = 2$.

The CVs recorded in Ar and CO_2 saturated electrolyte (Figure S5b) indicate the electrocatalytic response towards HER and eCO_2RR, with a significantly higher current in the presence of CO_2.

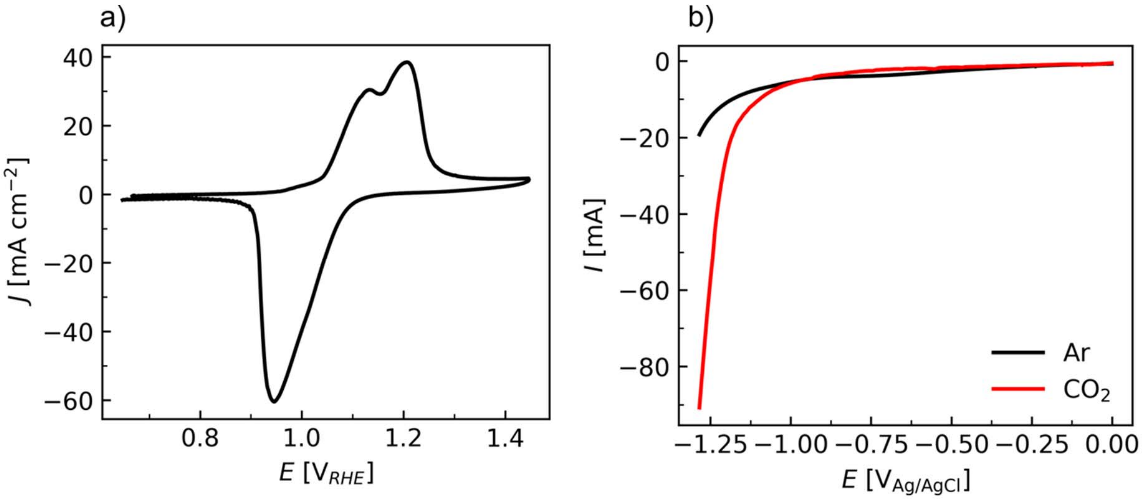

Figure S5 a) CV recorded with a scan rate of 20 mV s^{-1} in Ar saturated 0.1 M $KHCO_3$/ K_2CO_3 electrolyte on the redox-region. b) CVs recorded with a scan rate of 5 mV s^{-1} in Ar and CO_2 saturated electrolyte in the electrocatalytic region indicating HER and eCO_2RR, respectively.

S2. X-ray total scattering and pair distribution function analysis

X-ray total scattering experiments were performed at the P02.1 (λ = 0.2073 Å) beamline at PETRA III at the Deutsches Elektronen-Synchrotron (DESY) in Hamburg, Germany (Dippel *et al.*, 2015). Data were acquired on a VAREX XRD 4343CT detector (43 × 43 cm^2, pixel size of 150 × 150 μm^2) with a Q_{max} of 17.1 $Å^{-1}$, a sample-to-detector distance of 310 mm, a time resolution of 1 s, and a 1x1 mm^2 beam size.

The azimuthal integration and calibrations were performed with the pyFAI (V0.20) software (Kieffer *et al.*, 2020). PDFgetX3 (Juhás *et al.*, 2013) and xPDFsuite (Yang *et al.*, 2014) were used to obtain $S(Q)$, $F(Q)$, and $G(r)$ functions. A Q-range of 1.1–16.7 $Å^{-1}$ was used for the Fourier transformation of the *operando* data. In all cases, a R_{poly} = 0.9 was applied. Data were collected on a LaB6 standard to calibrate the exact geometry, in addition to the instrumental Q_{broad} and Q_{damp} parameters. Data modeling was performed with DiffPy-CMI (Juhas *et al.*, 2015) and NMF analysis with the nmfMapping application on the PDFitc platform (Thatcher *et al.*, 2022, Yang *et al.*, 2021). The PDFs were analyzed using the PCC in numpy (Harris *et al.*, 2020).

S2.1. The electrocatalytic CO_2 reduction reaction (eCO_2RR)

Stepped potential electrolysis was conducted in a CO_2 saturated 0.1 M $KHCO_3$/ K_2CO_3 electrolyte. The potential steps were: 0.0 V_{RHE}/ 300 s, -0.2 V_{RHE}/ 300 s, -0.4 V_{RHE}/ 300 s, -0.6 V_{RHE}/ 900 s, -0.8 V_{RHE}/ 900 s.

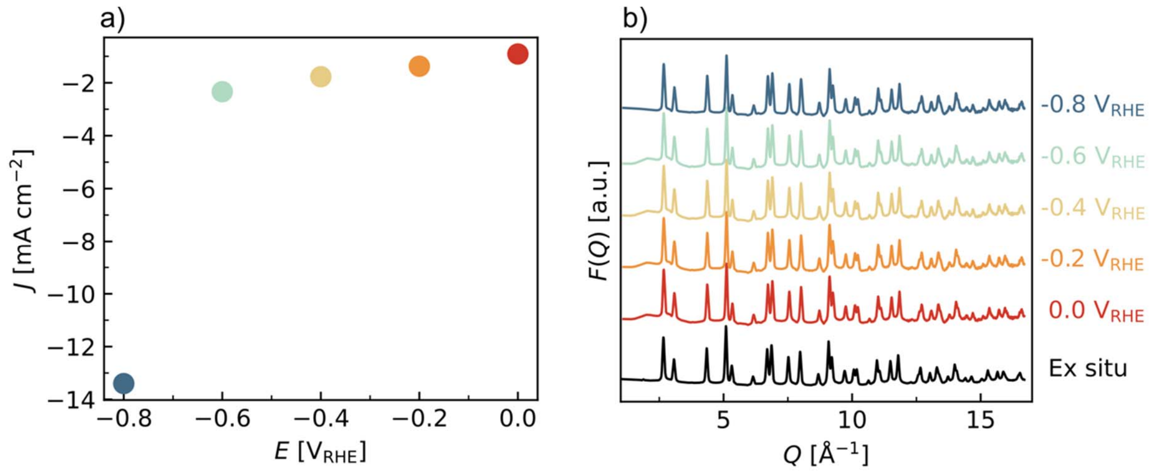

Figure S6 a) The current density at increasingly negative potentials, normalized to the surface area of the electrode (A = 5.06 cm^2). b) The total scattering function *F(Q)* at each potential. The frames during the last 180 s of each step were summed to obtain better statistics. The diffuse signal that gives rise to an amorphous background completely disappears at the most reductive potential of -0.8 V_{RHE}.

S2.1.1. Real space PDF refinements

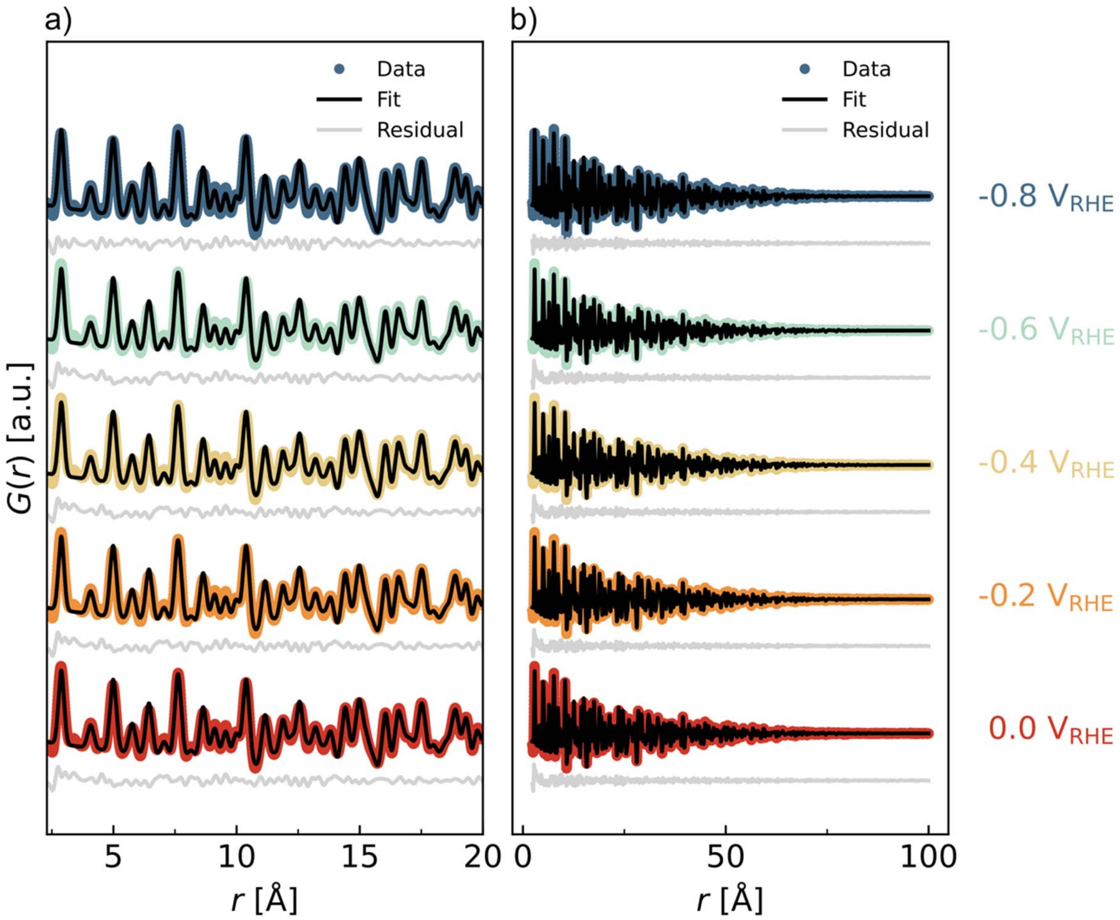

Figure S7 Refinements of the a) short-range PDFs and b) the long-range PDFs. Instrumental parameters were fixed based on refinement on a standard LaB_6 sample, as $Q_{damp} = 2.8350\times10^{-2}$ and $Q_{broad} = 9.0538\times10^{-3}$.

The structure of the catalyst under eCO_2RR conditions is compared with the structure of the catalyst under hydrogen evolution reaction (HER) conditions (*i.e.* in Ar saturated atmosphere at reducing potentials) – here just shown at one potential. In addition, the structure is compared with the *ex situ* structure.

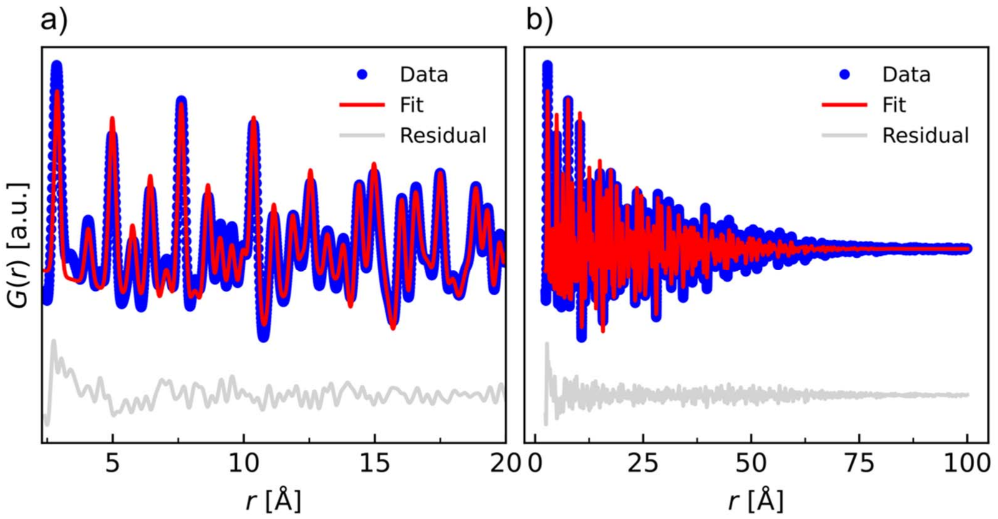

Figure S8 PDF refinements of the catalyst during the HER in Ar saturated electrolyte at -0.5 V_{RHE}. 180 frames of 1 s are summed. a) The short-range structure and b) the long-range structure.

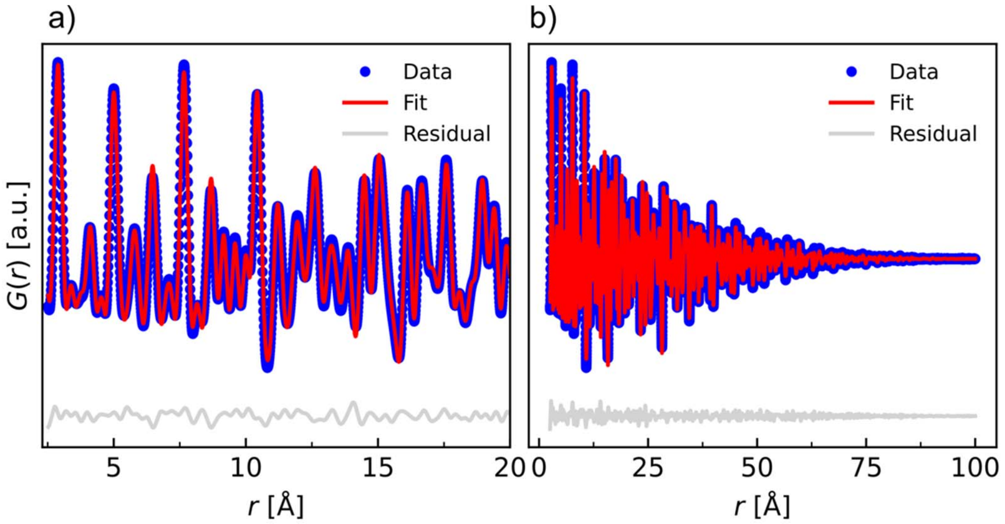

Figure S9 PDF Refinement of the *ex situ* catalyst. a) The short-range structure and b) the long-range structure.

Table S1 Refined parameters during the *operando* eCO_2RR and for the *ex situ* catalyst.

E [V_{RHE}]	Scale	*a* [Å]	B_{iso} [$Å^{-2}$]	Delta2	*size* [Å]	*Rw*
0.0	2.06(1)	4.07471(8)	0.74(4)	3.2(1)	142(2)	0.17
-0.2	2.07(1)	4.07469(8)	0.74(4)	3.2(1)	143(2)	0.17
-0.4	2.04(1)	4.07466.8)	0.74(4)	3.2(1)	142(2)	0.17
-0.6	1.92(1)	4.70462(8)	0.74(5)	3.2(1)	141(2)	0.18
-0.8	4.03(1)	4.07463(8)	0.72(2)	2.9(5)	150(1)	0.14
HER						
-0.5	1.65(1)	4.07047(1)	0.75(5)	3.2(1)	139(2)	0.21
Ex situ	0.31(1)	4.09268(5)	0.69(3)	4.1(7)	145(1)	0.10

The refined parameters of the catalyst under eCO_2RR conditions are comparable with the parameters under HER at similar potentials, within the errors.

S2.2. The Oxygen evolution reaction (OER)

Stepped potential electrolysis to increasingly oxidative potentials was conducted in an Ar saturated 0.1 M $KHCO_3$/ K_2CO_3 electrolyte for OER.

The potential steps were: 0.9 V_{RHE}/ 500 s, 1.2 V_{RHE}/ 900 s, 1.5 V_{RHE}/ 500 s, 1.7 V_{RHE}/ 1500 s. A longer holding time was selected at 1.2 V_{RHE} to account for the oxidation peak of Ag, and at 1.7 V_{RHE} as the onset potential of OER.

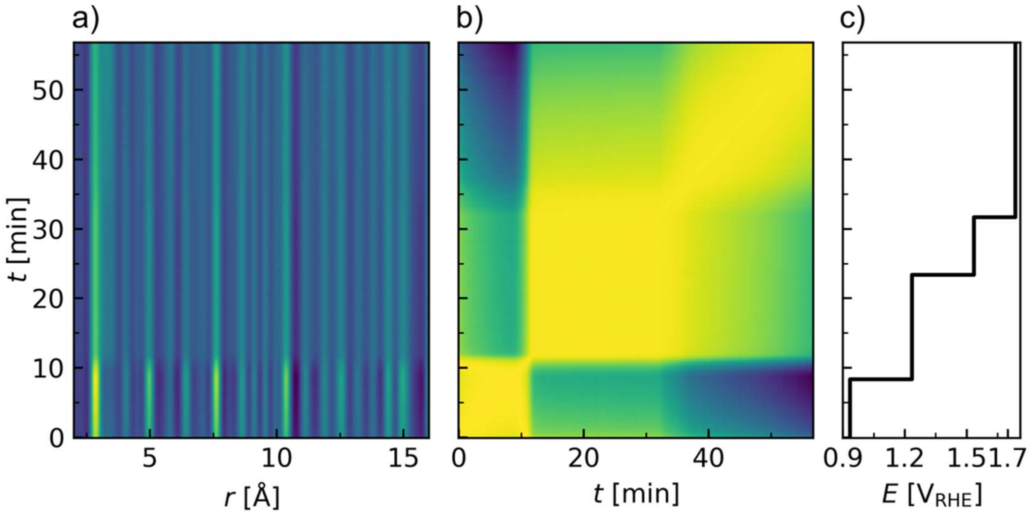

Figure S10 Time evolution of the PDFs during the oxidation experiment. a) Contour map of the local *r*-range of the PDFs (2-15.5 Å). b) PCC matrix between the time resolved PDFs. The color scale goes from blue, which represents the most dissimilar areas with a PCC of 0.75, to yellow, corresponding to a PCC of 1 c) The potential profile.

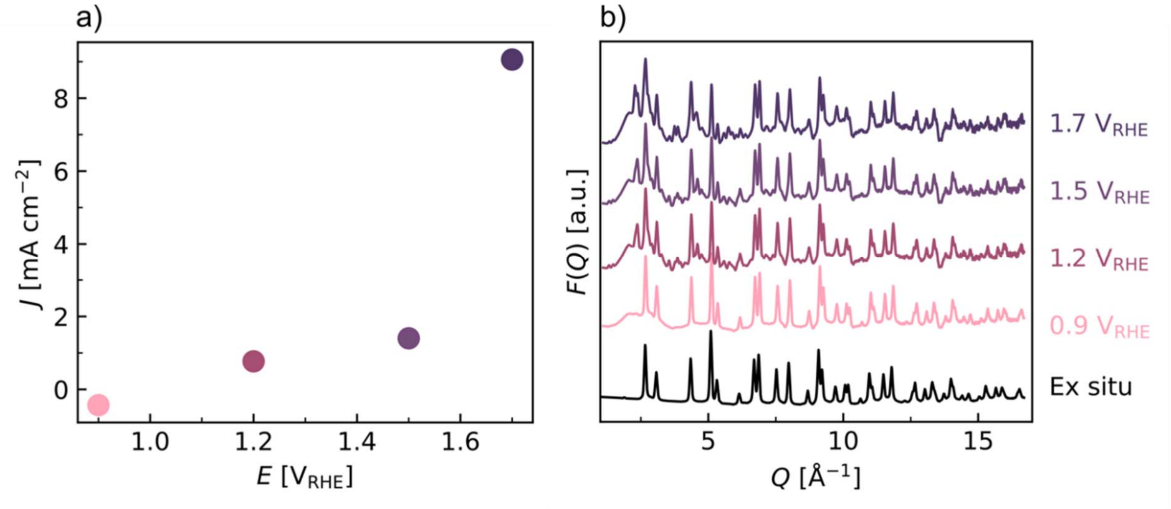

Figure S11 Stepped potential experiment conducted in an Ar-saturated 0.1 M $KHCO_3$/ K_2CO_3 electrolyte. a) The current density at increasingly oxidative potentials, normalized to the surface area of the electrode (A = 5.06 cm^2). b) The total scattering function *F(Q)* at each potential. The frames during the last 180 s of each step were summed to obtain better statistics. An increase in the amorphous content is observed as the potential increase as well as additional Bragg peaks.

S2.2.1. Refinement

Refinements of the oxidized catalyst were attempted against different silver carbonate and oxide compounds; however, they did not result in providing insights on the oxidized structure. This is expected to be due to the co-existence of 2-3 phases, and the many atomic contributions locally. Thus, in a short-range fit (1-15 Å) the information content is limited relative to the required variables and a long-range fit (1-100 Å) is too computationally heavy to perform with no good start-guesses. As a lot of information could be retrieved from model free analysis, real-space PDF refinements of the oxidized phase were not attempted further.

S2.2.2. Non-negative matrix factorization (NMF)

NMF analysis was performed on the local *r*-range from 2 Å to 15.5 Å. NMF analysis performed to the medium- to long range order, *i.e.* 2-30 Å and 2-100 Å, respectively, revealed the same trend (not included herein). Due to the restriction on the number of frames that can be uploaded to PDFitc, frames were summed to 50 s.

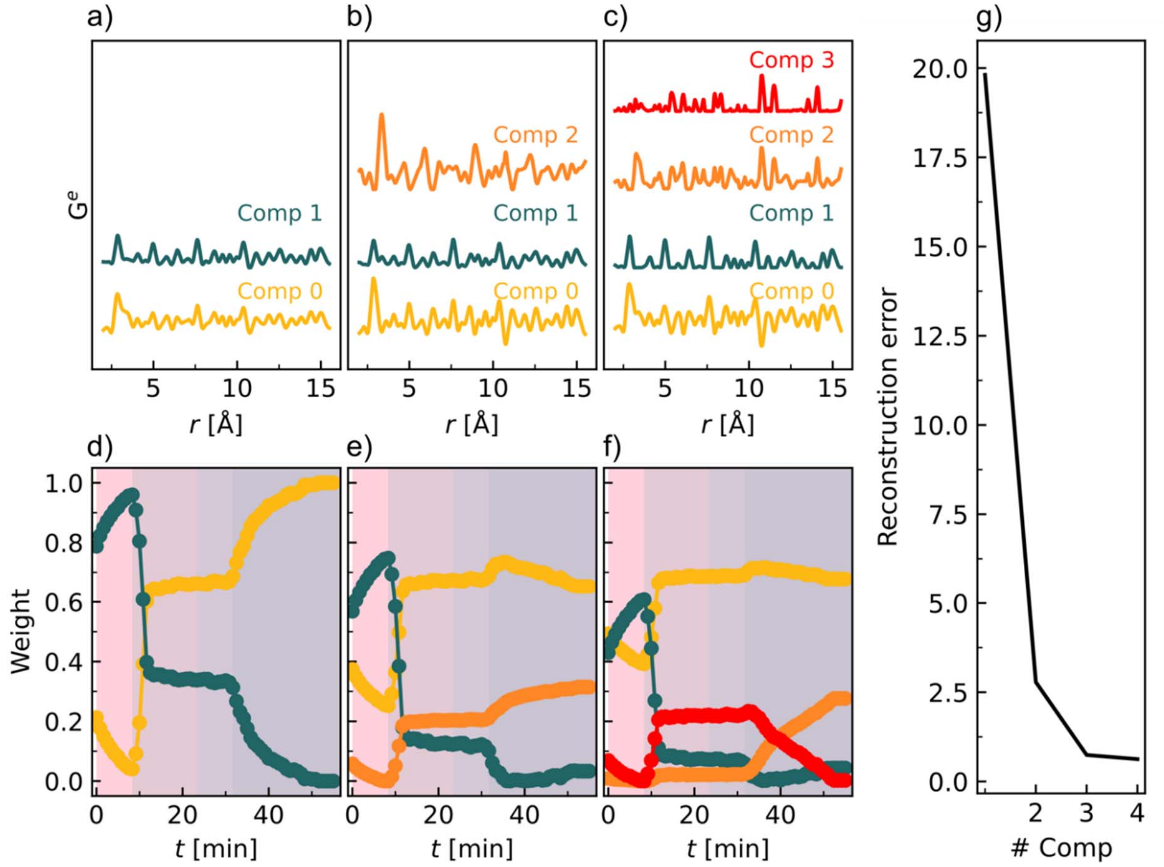

Figure S12 NMF analysis performed on the local *r*-range from 2 Å to 15.5 Å with a,d) two components, b,e) three components, and c,f) four components. a-c) The components and d-f) the evolution of the weight of the components. g) The reconstruction error *vs* the number of NMF components.

The attempt to track four or more NMF components results in NMF components that do not resemble real PDF data, *i.e.* the signals are clipped at low values.

S2.3. Cyclic Voltammetry

S2.3.1. Non-negative matrix factorization (NMF)

NMF analysis was performed on the local *r*-range from 2 Å to 15.5 Å. NMF analysis performed to the medium- to long range order, *i.e.* 2-30 Å and 2-100 Å, respectively, revealed the same trend (not included herein).

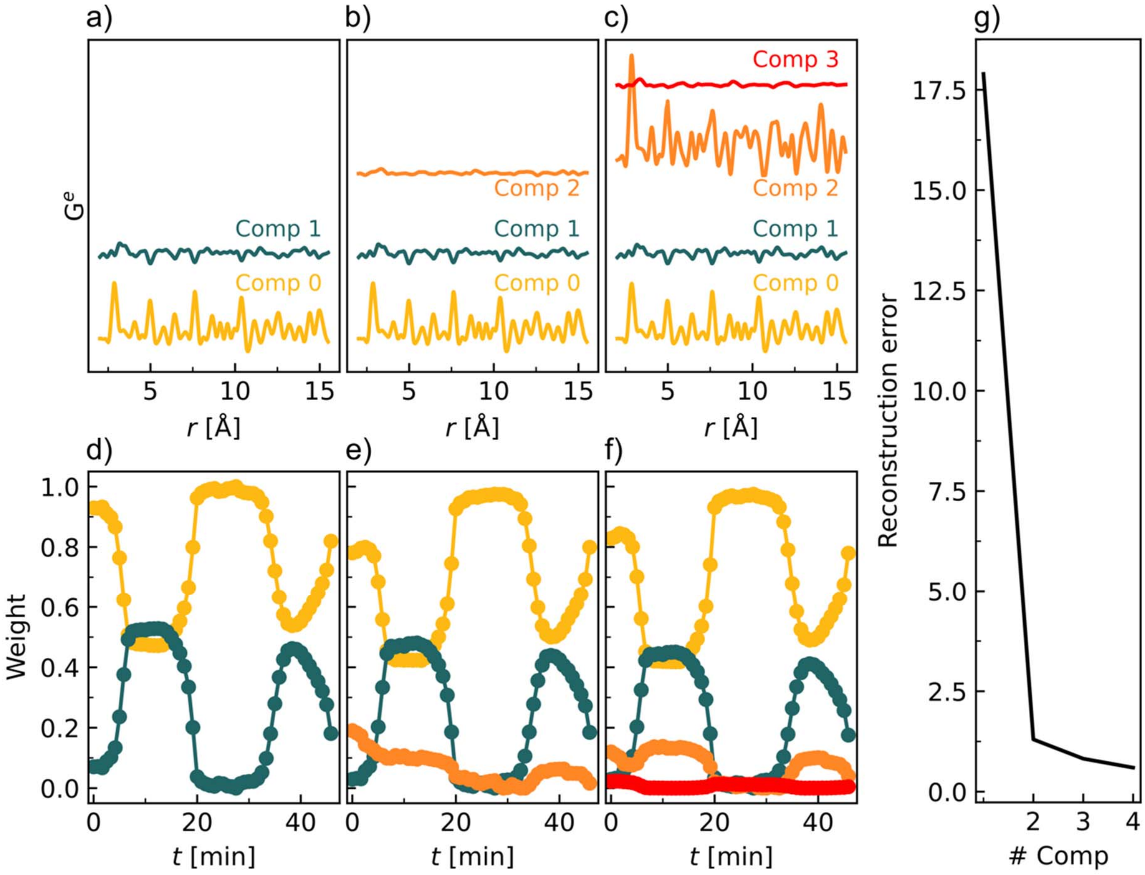

Figure S13 NMF mapping performed on the local *r*-range from 2 Å to 15.5 Å with a,d) two components, b,e) three components, and c,f) four components. a-c) The components and d-f) the evolution of the weight of the components. g) The reconstruction error *vs* the number of NMF components.

S2.4. A note on the background subtraction

The *operando* cell was constructed to contribute the smallest possible background. To obtain the PDF, precise background measurements must be performed at the different operating potentials. The total scattering signal from the *operando* cell with carbon paper as the working electrode (the support for the commercial Ag catalyst), the IrO_2 as the counter, a flow of electrolyte, and applied potential, was subtracted from the *operando* total scattering data with the Ag catalyst.

Despite the attempts to achieve a complete background correction, a diffuse signal, apparent as an amorphous background at ~2-4 $Å^{-1}$, is evident in all total scattering *operando* datasets in reciprocal space, especially in the oxidized catalysts. The diffuse signal cannot be ascribed with certainty to an amorphous/ disordered nature of the catalyst as soon as it is submerged in the electrolyte, or to the residual background. Multiple factors indicate that the amorphous content contains real structural information and is not just an artifact from insufficient background subtraction. These include the high likeness of the *operando* data under reducing conditions and the *ex situ* sample, the high-quality data and reliable refinement parameters, the trend in increasing amorphous content with more oxidizing potentials, as well as the diffuse signal in reciprocal space Fourier transforming to correlations in real space that can be assigned to Ag carbonate and Ag oxide peaks. However, it should be noted that carefully designing the background correction measurements is crucial for obtaining good PDF data, and even more so if an amorphous catalyst or very small nanoparticles are chosen as the pre-catalyst.

S3. X-ray diffraction (XRD)

X-ray diffraction was performed at both the P02.1 beamline ($\lambda = 0.2073$ Å) at PETRA III (Schökel *et al.*, 2021), and at the DanMAX beamline ($\lambda = 0.6199$ Å) at the MAX-IV laboratory in Sweden. At P02.1 the same detector and time resolution as in the total scattering experiments were utilized. At DanMAX, data were acquired on a DECTRIS PILATUS3 X 2M CdTe detector with a time resolution of 3 s, with 0.5 s of exposure and 2.5 s of latency to limit the beam damage. The beam was circular with a FWHM size of 500 μm and a high monochromaticity and energy resolution ($\Delta E/E \sim 10^{-4}$). Data were collected on a LaB_6 standard to calibrate the exact geometry and Rietveld refinements were performed using the Fullprof software (Rodríguez-Carvajal, 1993).

S3.1. 60 keV beam

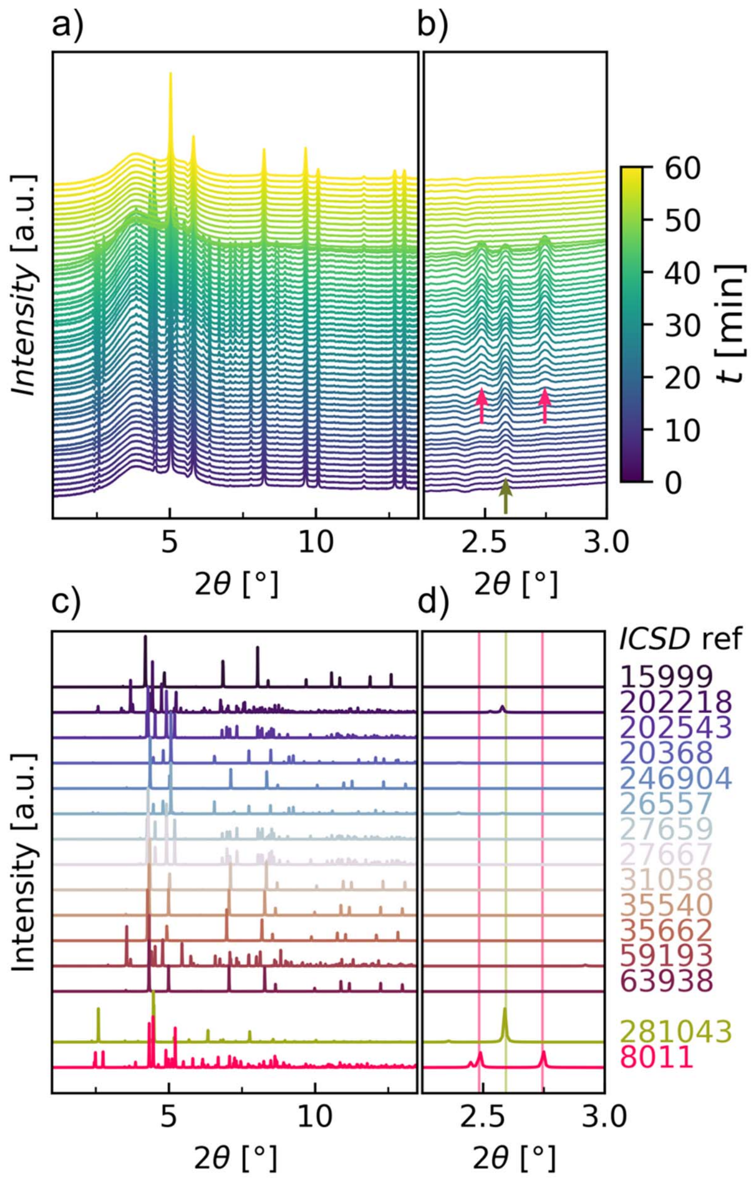

Figure S14 *Operando* XRD measured during prolonged anodic etching (45 min at 1.25 V_{RHE}) followed by reducing potentials (15 min at 0.85 V_{RHE}). a,b) Waterfall plot of the *operando* data. Every 60th frame is shown. The time scale of the evolution is represented on the colorbar to the right. a) The entire angular range and b) zoom on the low-angle region. c,d) Diffractograms of the most common different Ag oxides and carbonates. c) The entire angular range and d) zoom on the same low-angle region. The ICSD reference numbers are given to the right – see Table S2 for the chemical formula, space group symmetry and reference.

Table S2 List of phases compared in the fingerprint analysis in a S14.

All phases are recorded at T = 293 K and P = 101,325 Pa, except Ag_2CO_3/ HT (*ICSD* ref 281043) with T = 453 K.

ICSD ref	Hermann-Maguin Space Group	Struct. Form.	Reference
15999	P n -3 m S	Ag_2O_3	(Stehlik *et al.*, 1959)
202218	P 1 21/c 1	Ag_3O_4	(Standke & Jansen, 1987)
202543	P 1 21/c 1	Ag_2O_2	(Jansen & Fischer, 1988)
20368	P -3 m 1	Ag_2O	(Vereshchagin *et al.*, 1963)
246904	P n -3 m Z	Ag_2O	(Suzuki, 1960)
26557	P -3 1 m	Ag_6O_2	(Beesk *et al.*, 1981)
27659	P 1 21/c 1	AgO	(McMillan, 1960)
27667	C 1 2/c 1	AgO	(Salkind & Zeek, 1959)
31058	P n -3 m S	Ag_2O	(Niggli, 1922)
35540	P n -3 m S	Ag_2O	(Wyckoff, 1922)
35662	F -4 3 m	AgO	(Stehlík & Weidenthaler, 1959)
59193	F d d 2	Ag_2O_3	(Standke & Jansen, 1986)
63938	P n -3 S	Ag_2O	(Sagadevan *et al.*, 2023)
281043	P 3 1 c	Ag_2CO_3	(Norby *et al.*, 2002)
8011	P 1 21/m 1	Ag_2CO_3	(Masse *et al.*, 1979)

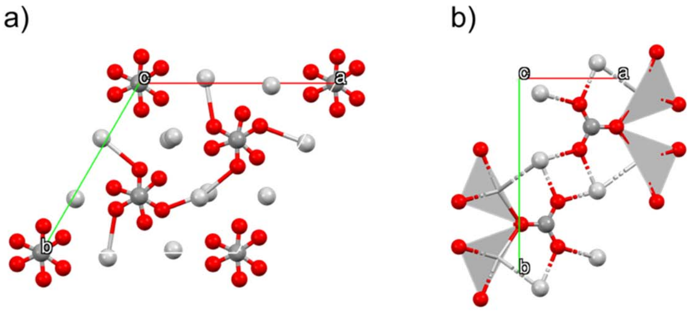

Figure S15 Crystal structures of the two Ag_2CO_3 phases generated with Mercury (Macrae *et al.*, 2020). View along *c* axis. a) Ag_2CO_3/ HT (space group P31c, *ICSD* ref: 281043) and b) Ag_2CO_3/ RT (space group P121/m1, *ICSD* ref: 8011).

S3.1.1. Sequential three-phase refinement

A sequential three-phase refinement is performed against the two Ag_2CO_3 phases and the Ag fcc structure. The refinement is performed with the Fullprof software (Rodríguez-Carvajal, 1993). Diffractograms are summed to a time resolution of 10 s. A glitch in the data between 3-4° is removed on all frames. Due to the difficulties in Fullprof of refining phases as their weight fraction goes to 0, the data were refined in two segments. The frames from 16.7 min to 60 min were refined against three phases. From 0 min to 15 min the data were refined against the two phases of Ag fcc and Ag_2CO_3/ HT. Attempts to include three phases below 16.7 min resulted in diverging values and singular matrices. 10 frames between 15 min and 16.7 min could not be refined due to this diverging nature in the range from two to three phases.

A manual background with 48 points is defined and refined. The peak shapes were modeled using the Thompson-Cox-Hastings pseudo-Voigt function. For all three phases, the scale and the unit cell parameters were refined. For the most reliable phase, Ag fcc, the isotropic thermal displacement parameter B_{iso} and the size parameter Y was refined as well, whereas for the two Ag_2CO_3 phases too much uncertainty in these resulted in their values being fixed at a standard value of 0.5 Å^2.

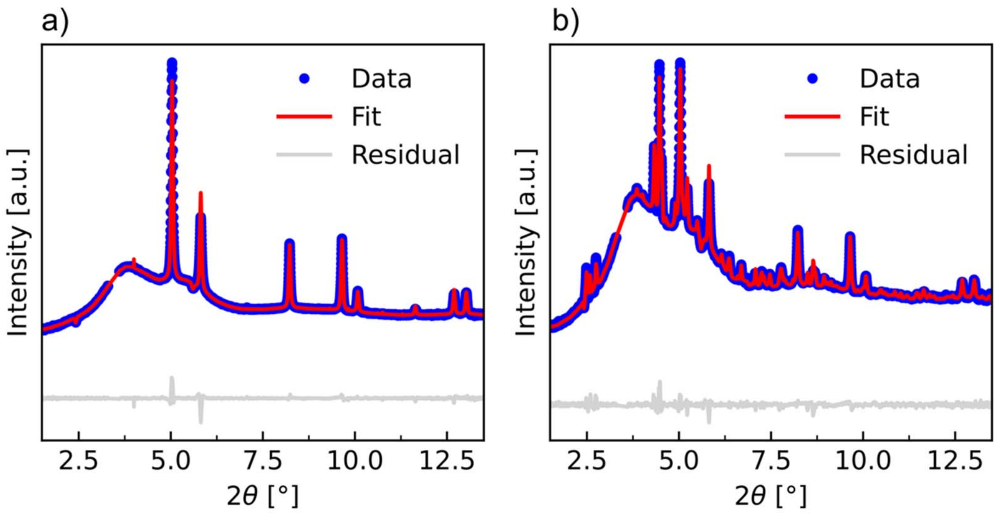

Figure S16 Refinements of a) the first frame and b) after 40 min.

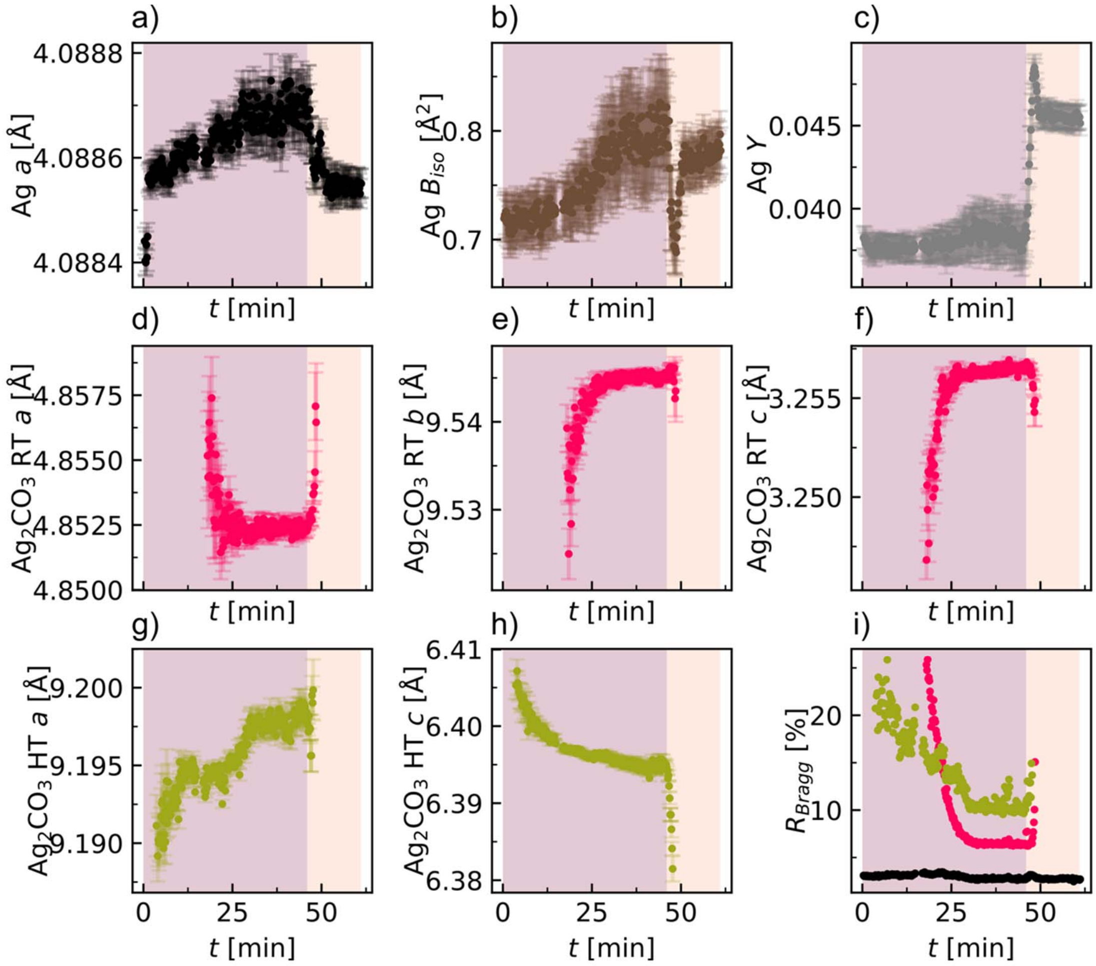

Figure S17 Refined parameters in the sequential three-phase refinement. Error bars directly from the refinements are shown in shaded colors. A filter is applied such that when the weight fraction of a phase is < 5%, parameters are not included, as the parameters become unreliable and the errors too big. a) The unit cell parameter *a* for the Ag fcc phase. b) The isotropic thermal displacement parameter B_{iso} for the Ag fcc phase. c) The size parameter *Y* for the Ag fcc phase. d,e,f) Unit cell parameters for the Ag_2CO_3/ RT phase, *a, b,* and *c* axes, respectively. g,h) The unit cell parameters for the Ag_2CO_3/ HT phase, *a* and *c* axes, respectively. i) The goodness-of-fit parameter R_{Bragg}. Sufficient statistics are needed before good values are obtained.

S3.1.2. Non-negative matrix factorization

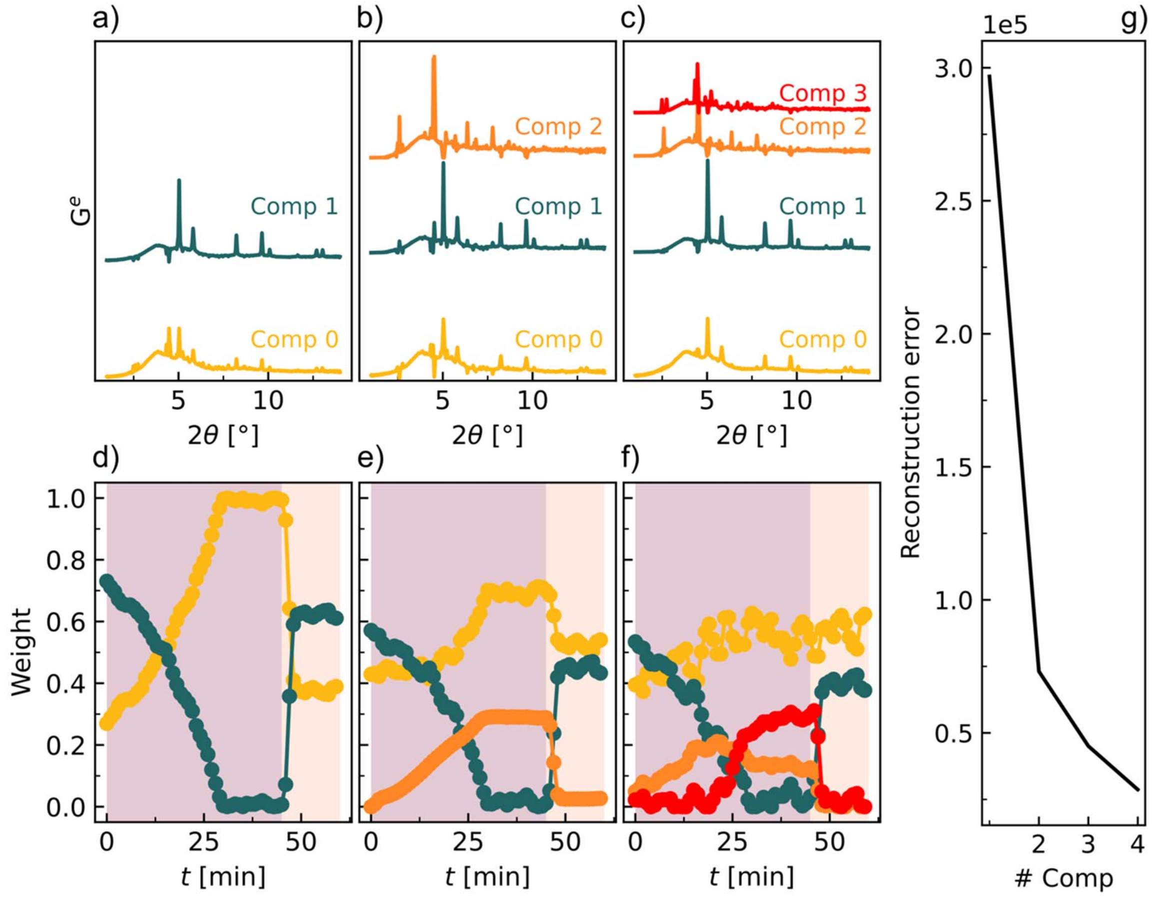

Figure S18 NMF mapping performed on the entire angular range with a,d) two components, b,e) three components, and c,f) four components. a-c) The components and d-f) the evolution of the weight of the components. g) The reconstruction error *vs* the number of NMF components.

S3.2. 20 keV beam

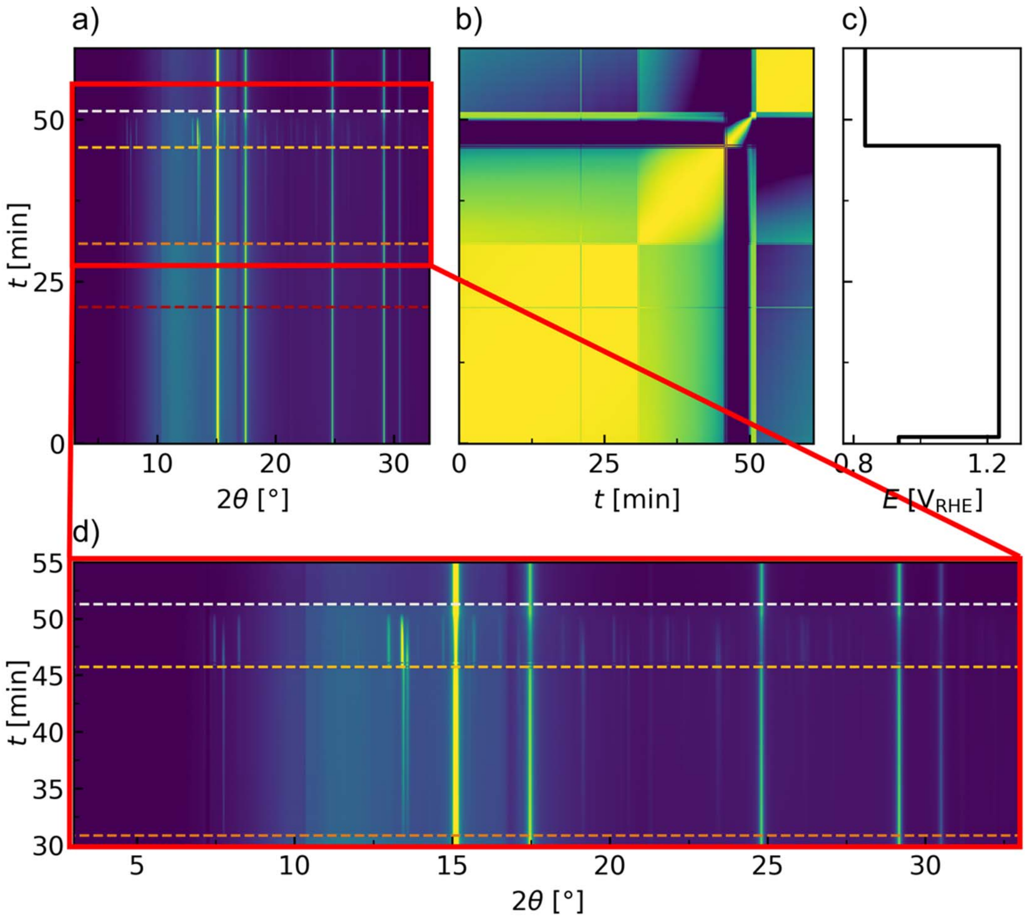

Figure S19 *Operando* XRD measured during prolonged anodic etching (45 min at 1.25 V_{RHE}) followed by reducing potentials (15 min at 0.85 V_{RHE}). a) Contour map over the time evolution of XRD data during the experiment. The dashed lines represent changes in the sample position. b) PCC matrix between the time-resolved XRD data, indicating structurally distinct regions. The color scale goes from blue, which represents the most dissimilar areas with a PCC of 0.76, to yellow, corresponding to a PCC of 1. c) Pulsed potential profile. d) Zoom on the sample position changes.

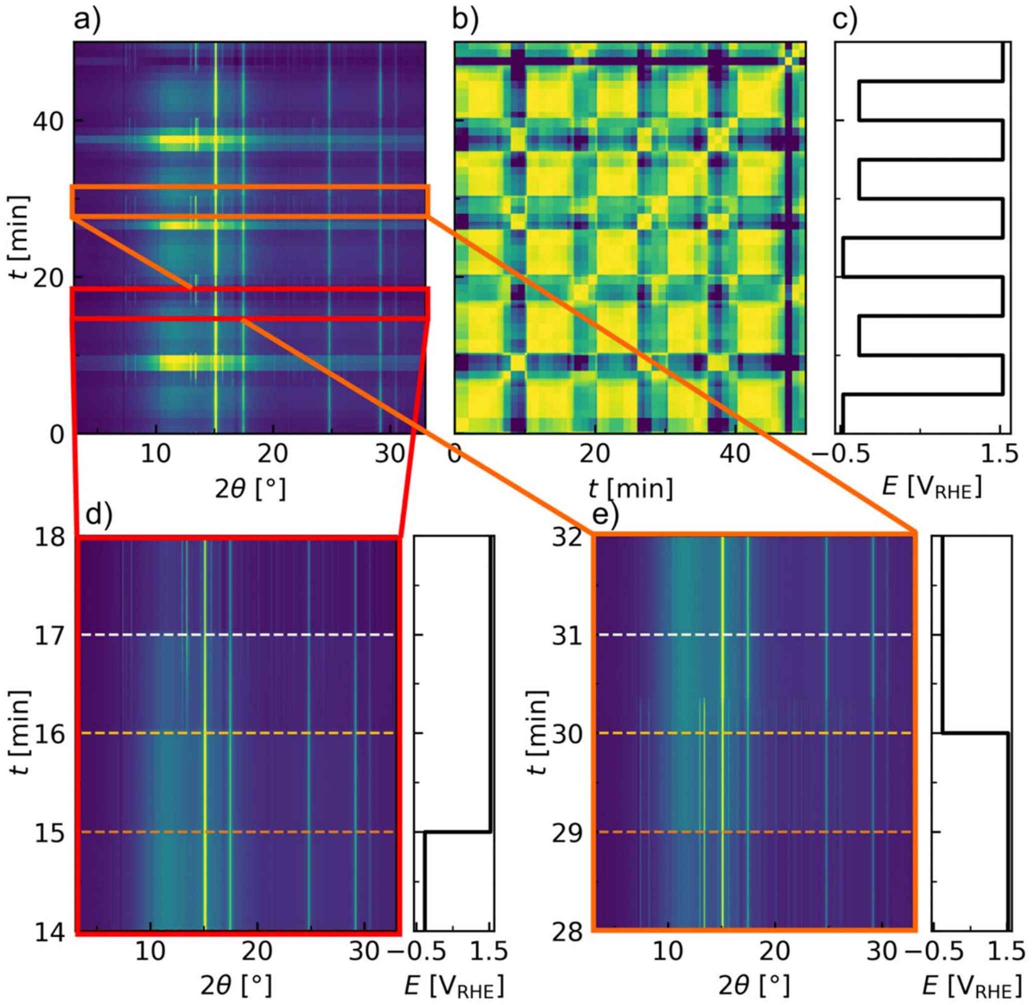

Figure S20 *Operando* XRD measured during a pulsed potential experiment with a switch between cathodic and anodic potential every 5 min. 50 different positions on the sample were scanned, with a new position every 60 s. a) Contour map over the time evolution of XRD data during the experiment. b) PCC matrix between the time resolved XRD data, indicating structurally distinct regions. The color scale goes from blue, which represents the most dissimilar areas with a PCC of 0.42, to yellow, corresponding to a PCC of 1. c) Pulsed potential profile. d) Zoom on a switch from cathodic to anodic potential, dashed lines represent changes in position. e) Zoom on a switch from anodic to cathodic potential.

A clear difference is observed when the potential is working with the beam (reductive/ cathodic) vs against the beam (oxidative/ anodic). The oxidation is only visible when the position is moved to a fresh spot, while the reduction is visible in between positions.

S3.2.1. Non-negative matrix factorization

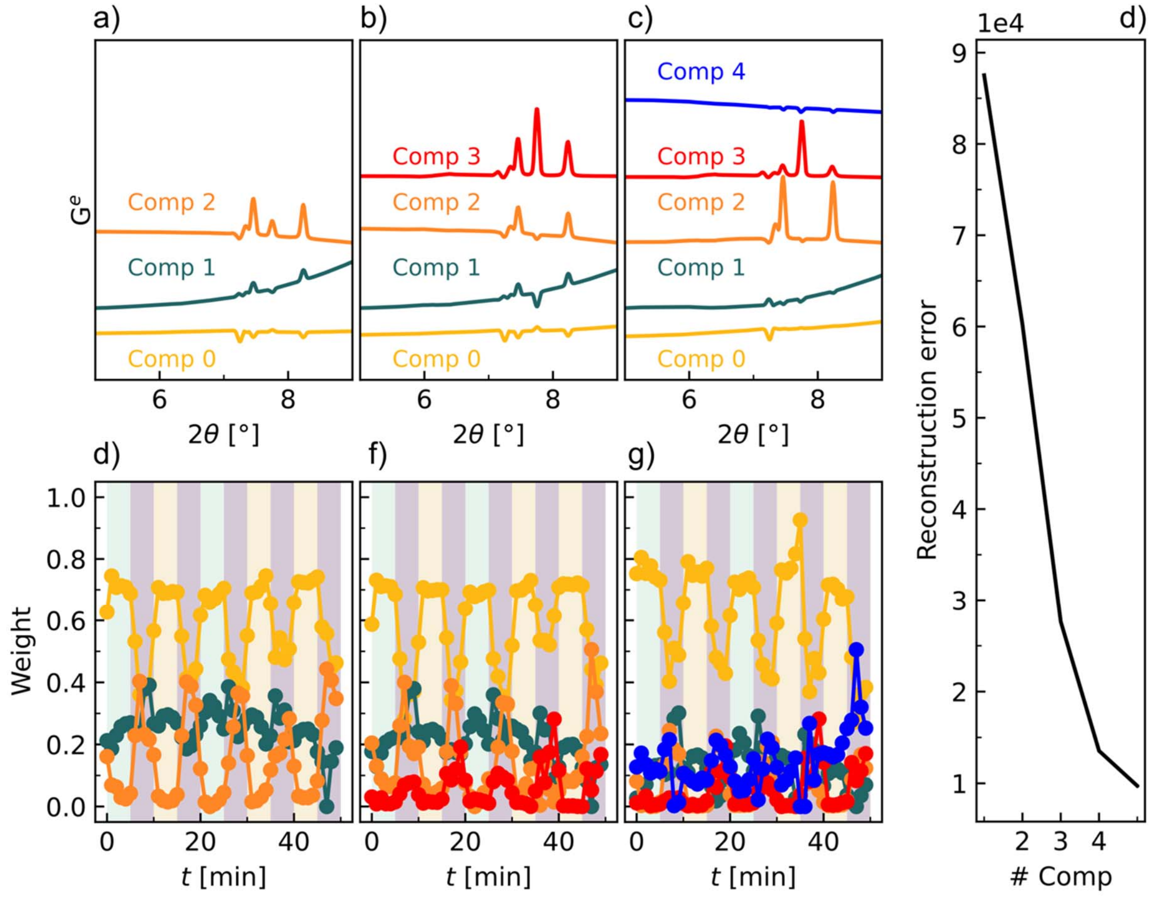

Figure S21 NMF mapping performed on the low-angle range from 5-9° with a,d) two components, b,e) three components, and c,f) four components. a-c) The components and d-f) the evolution of the weight of the components. g) The reconstruction error *vs* the number of NMF components.

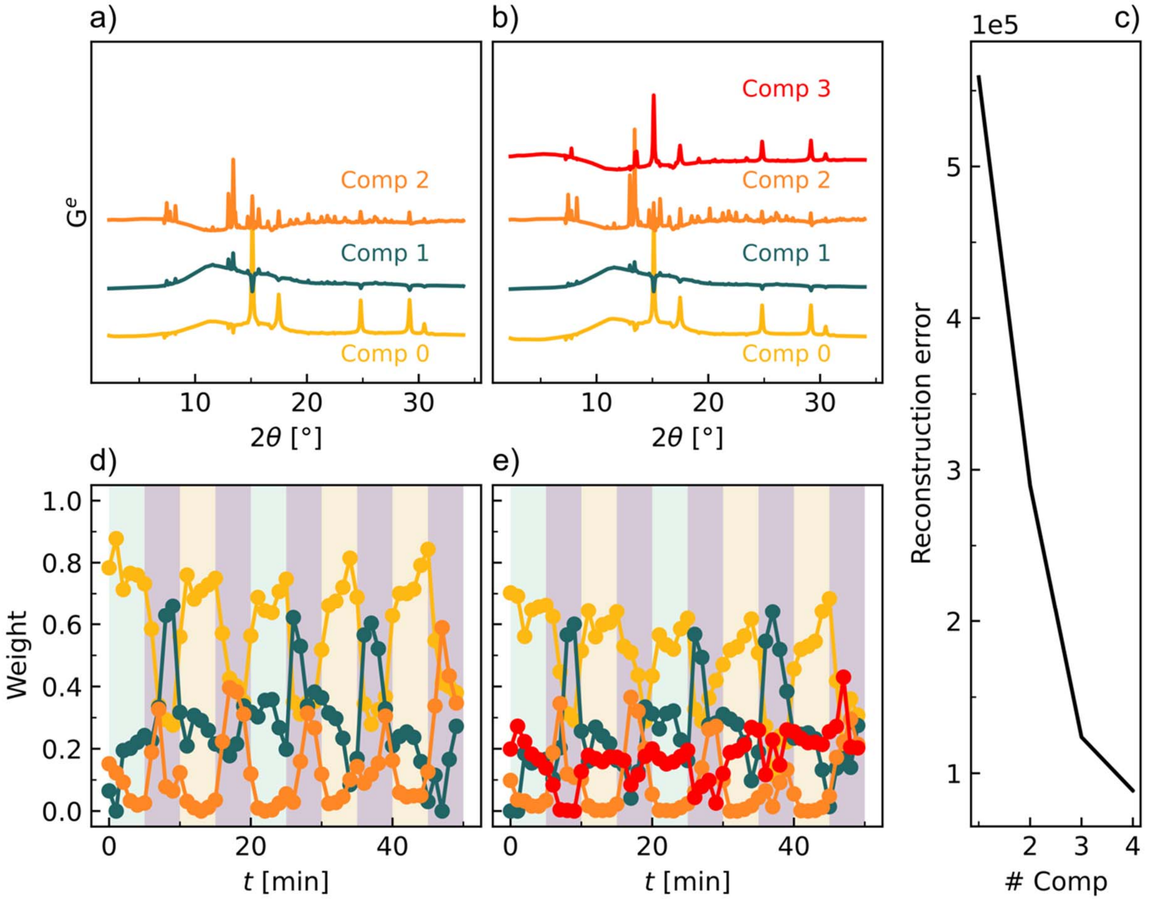

Figure S22 NMF mapping performed on the entire angular range with a,d) three components and b,e) four components. a,b) The components and d,e) the evolution of the weight of the components. c) The reconstruction error *vs* the number of NMF components.

S4. Scanning electron microscopy (SEM)

Scanning electron microscopy (SEM) is performed on a FEI-Nova Nano SEM 600 under high-vacuum conditions in field immersion mode through a lens detector (TLD). An accelerating voltage of 5.00 kV was utilized. The electrodes were immobilized on aluminum supports with conductive carbon tape.

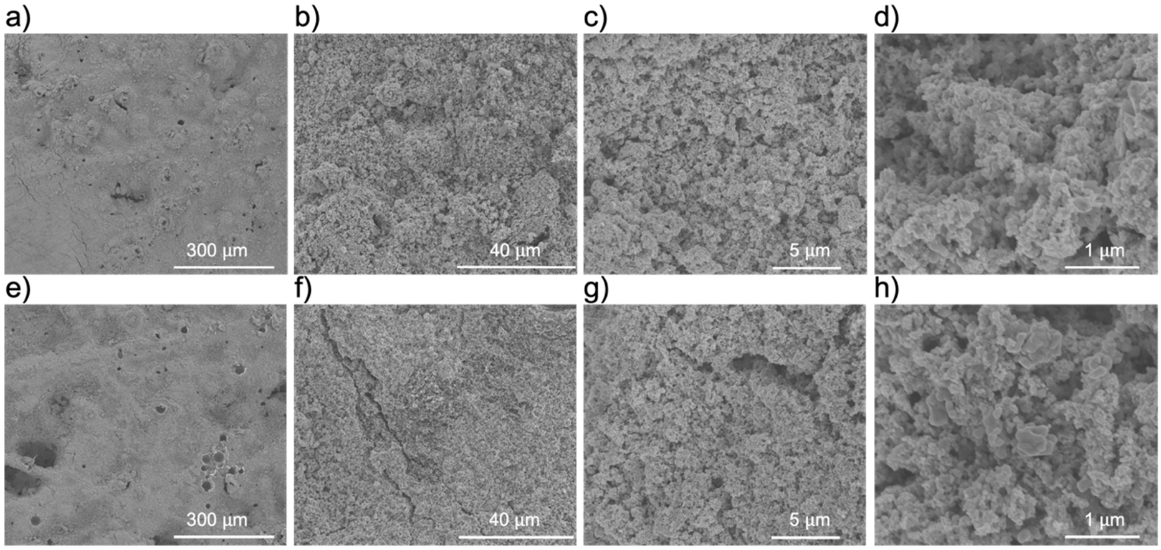

Figure S23 SEM images on the electrode a-d) before and e-h) after the prolonged anodic etching experiment with a 45 min pulse at 1.25 V_{RHE} and 15 min at 0.85 V_{RHE}. The scalebars are a,e) 300 µm, b,f) 40 µm, c,g) 5 µm, and d,h) 1 µm.

Table S3 Quantification of elements measured with SEM energy dispersive X-ray spectroscopy (EDS).

Carbon is both expected from the support (carbon tape) and if carbonate is present. Fluorine is expected from the binder used in preparation of the electrode. It is noted that there are no traces of potassium from the electrolyte left after the electrolysis experiment.

Element (edge)	At% before	At% after
C (K)	78	81
F (K)	7	7
Ag (L)	15	12

S5. Inductively coupled plasma – optical emission spectroscopy (ICP-OES)

Solid samples were transferred in quartz tubes and placed in a microwave oven, Anton Paar MW3000. 5 mL of concentrated nitric acid was added, and after sealing the quartz tubes the samples were dissolved by applying 1400 W microwaves for 20 minutes.

The content of the quartz tubes was transferred to sterile cups and 100 µL of internal standard (Y) were added. Finally, the volume of the samples was adjusted with MilliQ water (100 g), resulting in a nitric acid concentration of 5% (v/v).

5 mL of the liquid sample was transferred to sterile cups and the volume was adjusted to 100 mL with a solution of nitric acid (5 % v/v).

The measurements were performed with a Perkin Elmer Optima 4300DV, equipped with a Peek Mira mist nebulizer, a Scott spray chamber, and Quartz torch (tulip) with an Alumina injector 2.0 mm i.d. The instrument presents a Dual View (DV) mode; therefore, the plasma could be viewed both axially and radially.

S5.1. Electrode

Three samples of the commercial silver electrocatalyst (area approximately 0.317 cm^2), and with masses of 3.0 mg, 3.2 mg, and 3.0 mg, were analyzed. The amount of silver detected was 1.4 ± 0.1 mg cm^{-2}.

S5.2. Electrolyte

The electrolyte from the working electrode reservoir was saved after the anodic pulse experiment performed at 60 keV with 45 min at 1.25 V_{RHE} followed by 15 min at 0.85 V_{RHE}. The electrolyte was brought home and analyzed with ICP-OES.

Only Ag traces were detected, thus a complete separation of the two half cells with the Ag working electrode and the IrO_2 counter electrode was successful, as no Ir leached to the side of the working electrode.

The Ag concentration in mg L^{-1} electrolyte is determined from 3 experiments as: 0.0883(5) mg L^{-1}.

With a reservoir volume of 500 mL, this corresponds to an absolute amount of Ag dissolved of 0.0442(3) mg. The loading of the electrode is 1.4 mg cm^{-2} and with a surface area of 5.06 cm^2, the dissolved Ag corresponds to 0.6% of the Ag loaded onto the electrode.

S6. Multimodal X-ray absorption spectroscopy and diffraction

Experiments were performed at the Balder beamline at the MAX-IV laboratory in Sweden, with a high photon flux (10^{12} photons/s) and high resolution ($\Delta E/E \sim 2\times10^{-4}$) (Klementiev *et al.*, 2016). X-ray absorption spectroscopy was performed at the K edge of silver (25514 eV) in transmission mode using a double Si(111) monochromator. Spectra were recorded covering both the X-ray absorption near edge structure (XANES) and the extended X-ray absorption fine structure (EXAFS) region in an energy range of -300 to + 800 eV relative to the edge.

Simultaneously, XRD data was recorded on a 2D EIGER 1M detector at an energy just below the absorption edge of silver at 25201 eV, in a Q-range range of 1.15-6.13 Å^{-1}.

Data were recorded with a time resolution of 4 s, with a 3 s scan time for the XAS measurements and a 1 s exposure for the XRD, and a beam size of 100×100 μm^2.

XAS data were processed with the Larch python package (Newville, 2013). The pre-edge line was fitted from -195 eV to -65 eV and the post-edge from 5 eV to 800 eV in reference to the edge position.

S6.1. X-ray absorption near edge structure (XANES)

As the formation of the carbonate phase was unknown prior to the beamtimes, no carbonate standard was brought. Thus, the standard spectrum from Ag_2CO_3 data published in MDR XAFS DB was used for XANES analysis (Ishii *et al.*, 2021, Ishii *et al.*, 2023). The spectrum was aligned to the measured Ag foil and Ag oxides.

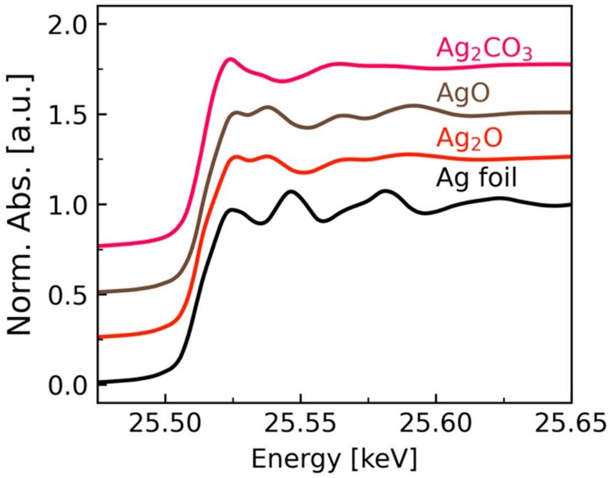

Figure S24 The XANES region for the three standards of metallic Ag foil (black), Ag_2O (red), and AgO (brown), representing the oxidation states of 0, +I, and +II, respectively, as well as Ag_2CO_3 (pink). Only small differences are observed between the two oxide phases in the XANES region.

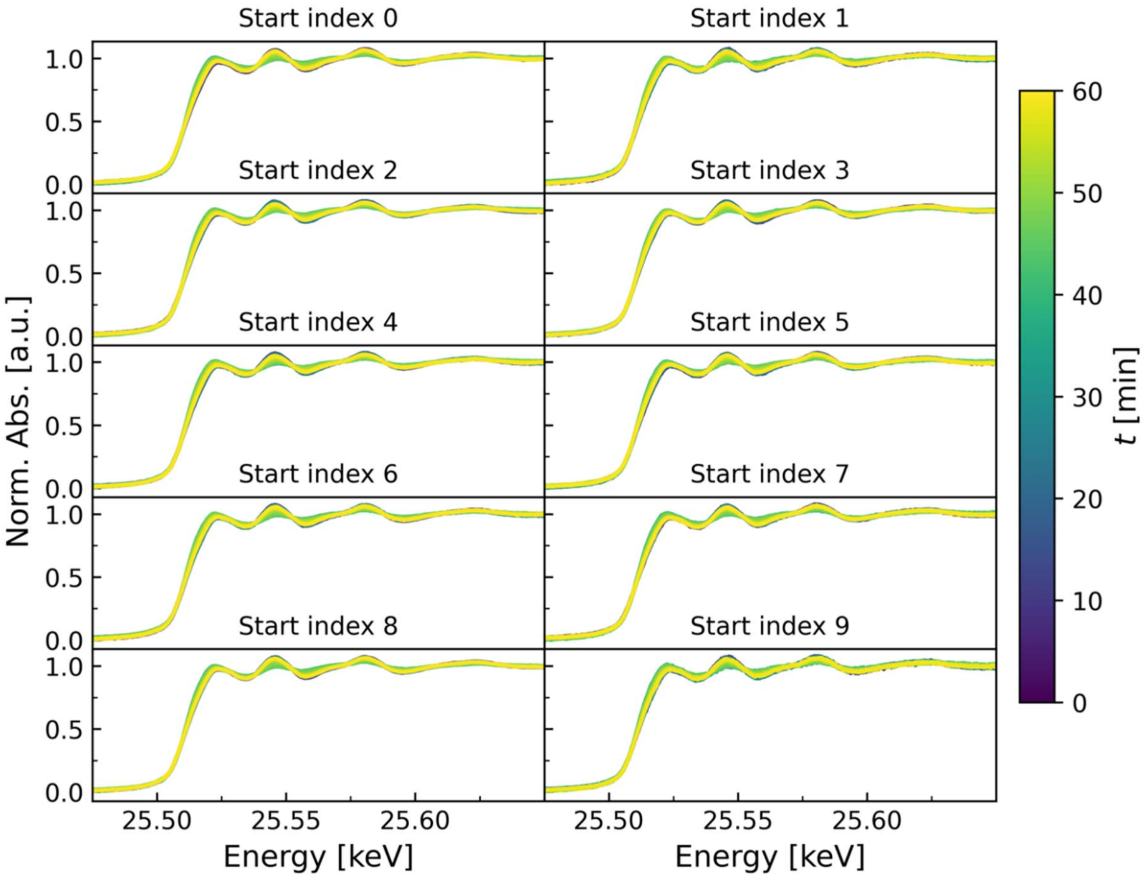

Figure S25 The XANES region probed at 10 different positions (index 0-9). The time evolution is represented by overlaying the spectra recorded at each position, from the start of the experiment (blue) to the end (yellow). The colorbar to the right represents the time.

S6.1.1. Linear combination analysis (LCA)

Linear combination analysis (LCA) fitting was conducted over the energy range -30 to 80 eV relative to the edge. The edge energy E_0 was fixed.

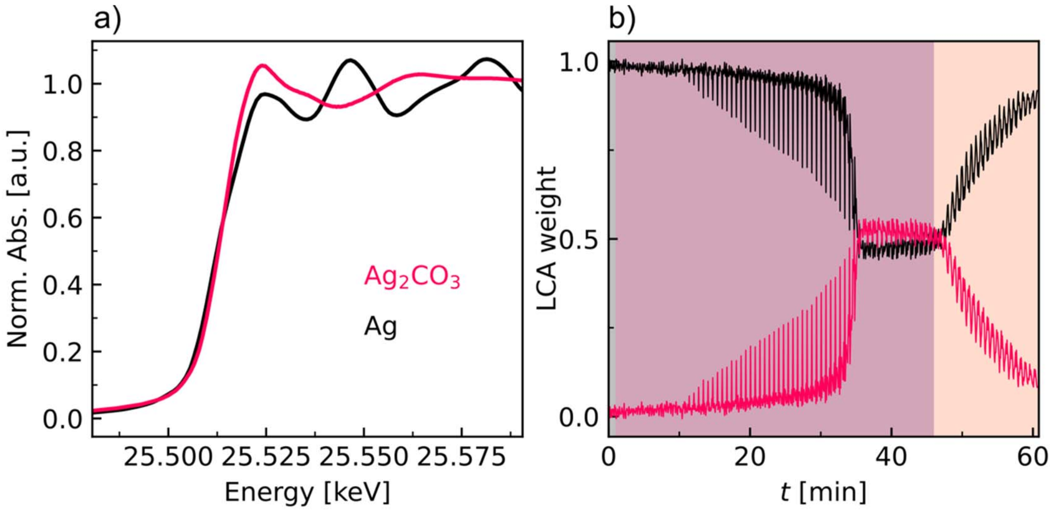

Figure S26 LCA fitting against the standard reference spectra of a Ag foil and Ag_2O. a) The XANES region of the two standard reference spectra, Ag (in black) and Ag_2CO_3 (in pink). b) The weight of each component, throughout the *operando* experiment. The lavender region indicates a potential of 1.25 V_{RHE} and the pale-yellow the cathodic potential of 0.85 V_{RHE}.

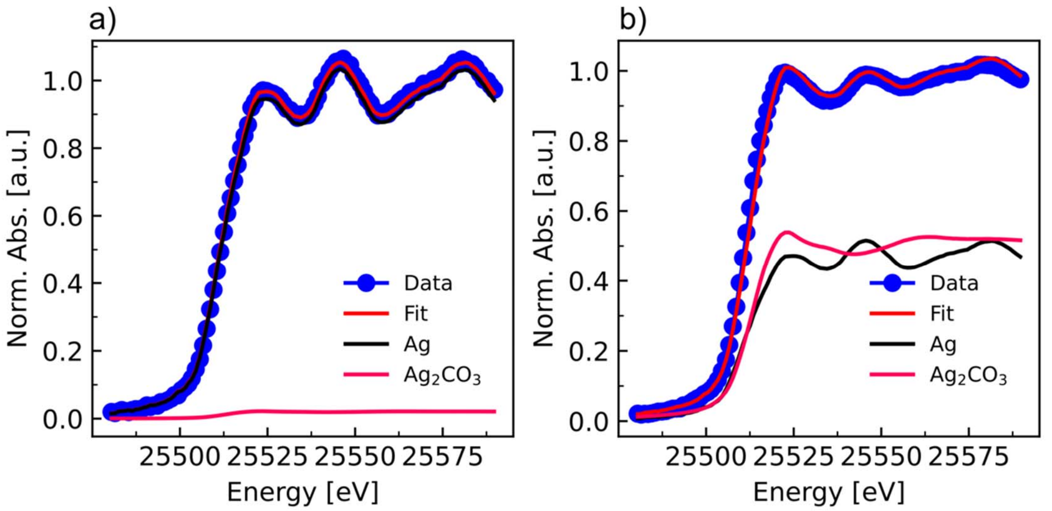

Figure S27 Results of the LCA fitting on a) the starting structure and b) after 37 min at a potential hold of 1.25 V_{RHE}. The chi-squared values are 0.005 and 0.032 respectively.

S6.2. Extended X-ray absorption fine structure (EXAFS)

The EXAFS data were re-binned in *k*-space.

S6.2.1. *k*-space

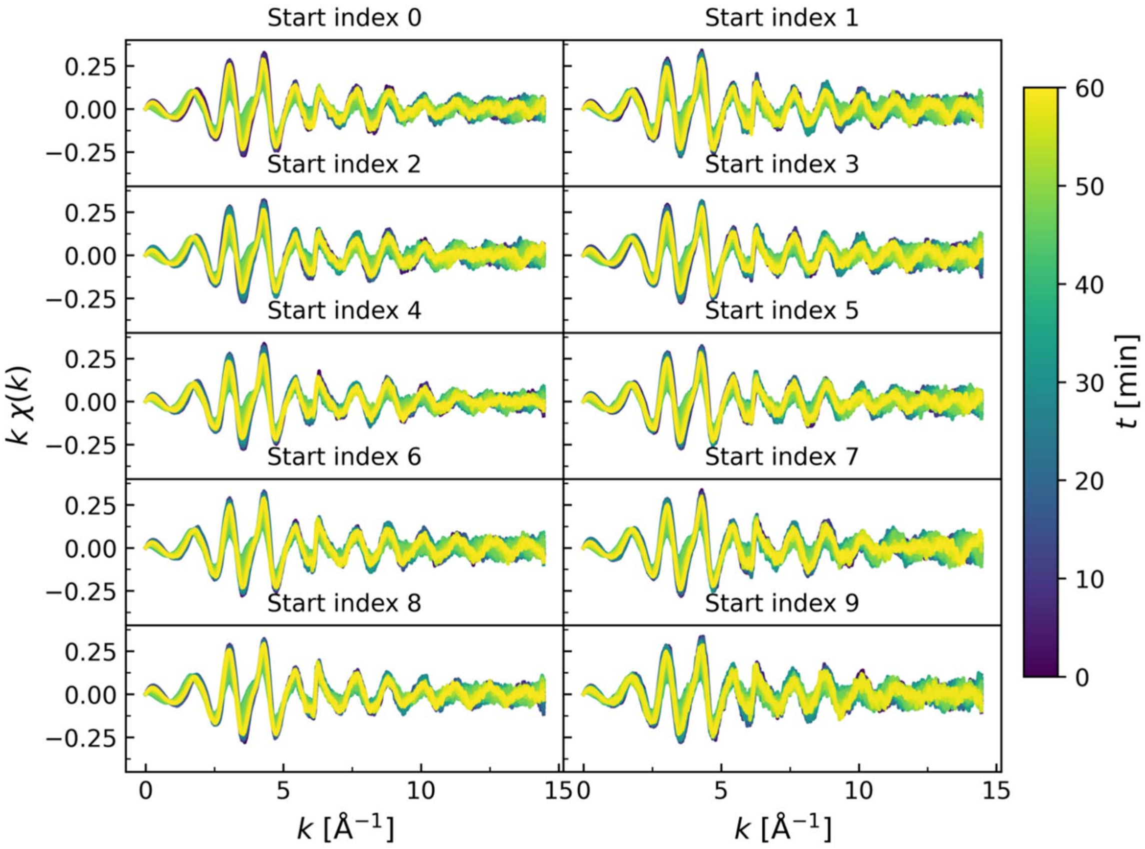

Figure S28 The *k*-weighed χ(*k*) functions probed at 10 different positions (index 0-9). The time evolution is represented by overlaying the spectra recorded at each position, from the start of the experiment (blue) to the end (yellow). The colorbar to the right represents the time.

S6.2.2. *R*-space

The Fourier transform was performed in a *k*-range from 3-13 Å^{-1}.

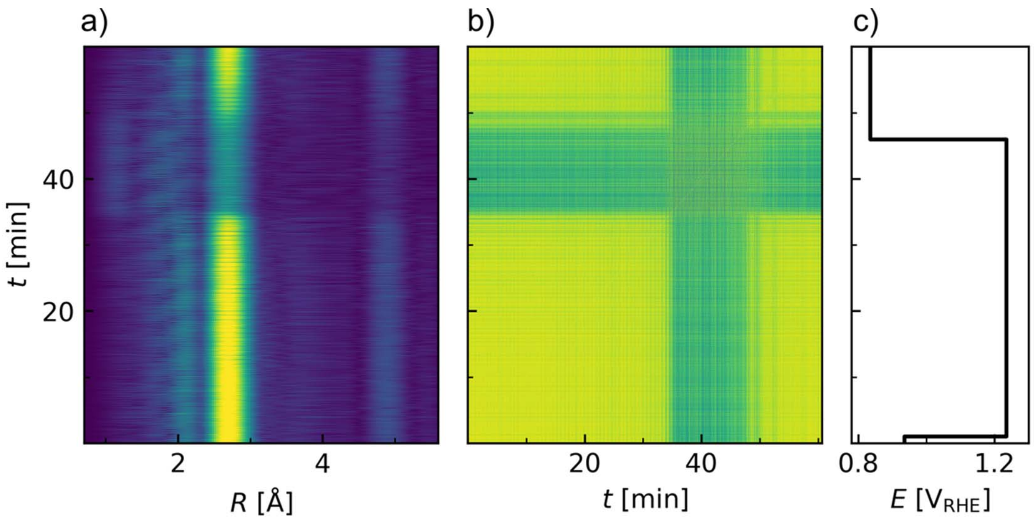

Figure S29 Evolution of the modulus of the k^2 weighed $\chi(R)$ functions during the *operando* experiment. The Fourier transform is performed in a *k*-range from 3-13 Å^{-1}, here shown as k^2 weighed data. a) Contour map of the time evolution of the k^2 weighed $\chi(R)$. b) The PCC matrix for the EXAFS signal in *R*-space. The color scale goes from blue, which represents the most dissimilar areas with a PCC of 0.65, to yellow, corresponding to a PCC of 1. c) The pulsed potential profile.

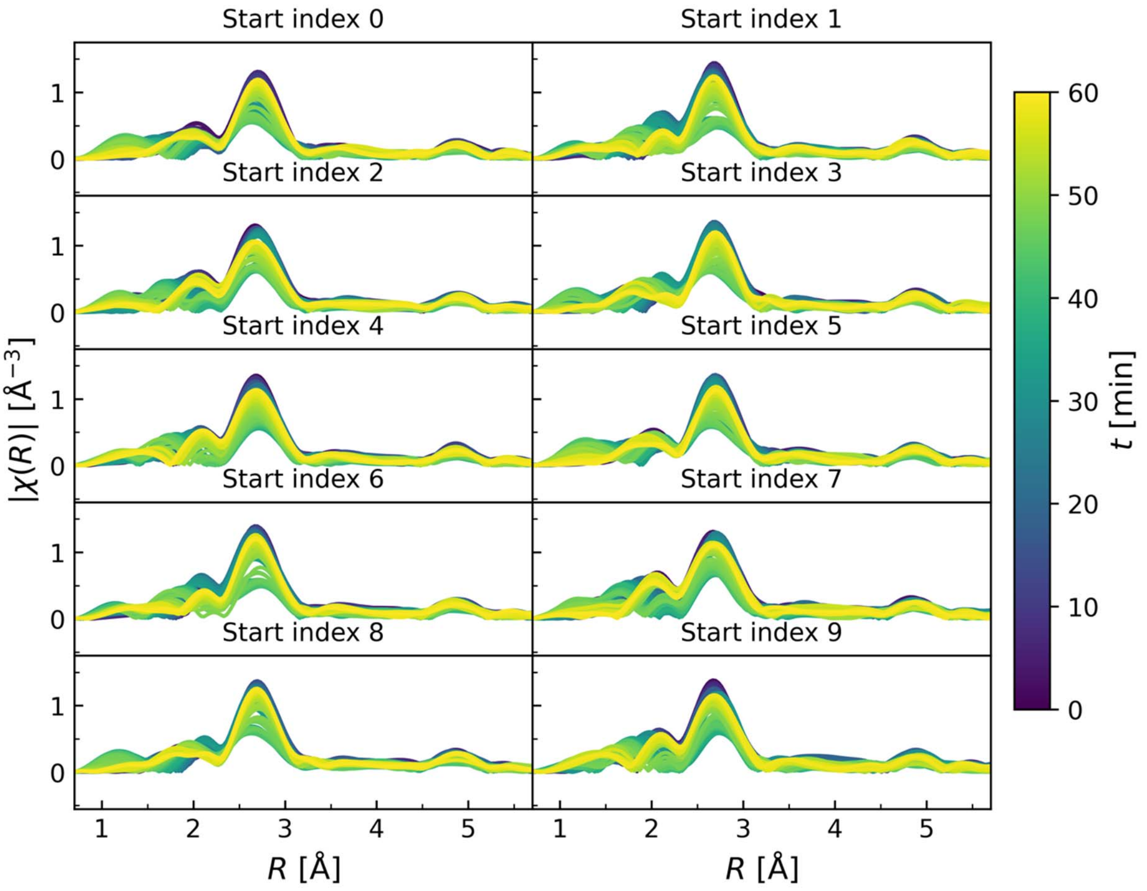

Figure S30 The modulus of the k^2 weighed $\chi(R)$ functions probed at 10 different positions (index 0-9). The time evolution is represented by overlaying the spectra recorded at each position, from the start of the experiment (blue) to the end (yellow). The colorbar to the right represents the time.

References

Beesk, W., Jones, P. G., Rumpel, H., Schwarzmann, E. & Sheldrick, G. M. (1981). *J Chem Soc Chem Comm*, 664-665.

Dippel, A. C., Liermann, H. P., Delitz, J. T., Walter, P., Schulte-Schrepping, H., Seeck, O. H. & Franz, H. (2015). *J. Synchrotron Rad.* **22**, 675-687.

Harris, C. R., Millman, K. J., van der Walt, S. J., Gommers, R., Virtanen, P., Cournapeau, D., Wieser, E., Taylor, J., Berg, S., Smith, N. J., Kern, R., Picus, M., Hoyer, S., van Kerkwijk, M. H., Brett, M., Haldane, A., del Río, J. F., Wiebe, M., Peterson, P., Gérard-Marchant, P., Sheppard, K., Reddy, T., Weckesser, W., Abbasi, H., Gohlke, C. & Oliphant, T. E. (2020). *Nature* **585**, 357-362.

Ishii, M., Nagao, H., Tanabe, K., Matsuda, A. & Yoshikawa, H. (2021). *Materials Data Repository,*, National Institute for Materials Science.

Ishii, M., Tanabe, K., Matsuda, A., Ofuchi, H., Matsumoto, T., Yaji, T., Inada, Y., Nitani, H., Kimura, M. & Asakura, K. (2023). *Science and Technology of Advanced Materials: Methods* **3**, 2197518.

Jansen, M. & Fischer, P. (1988). *J. Less-Common Met.* **137**, 123-131.

Juhás, P., Davis, T., Farrow, C. L. & Billinge, S. J. L. (2013). *J. Appl. Crystallogr.* **46**, 560-566.

Juhas, P., Farrow, C. L., Yang, X. H., Knox, K. R. & Billinge, S. J. L. (2015). *Acta Crystallogr. A* **71**, 562-568.

Kieffer, J., Valls, V., Blanc, N. & Hennig, C. (2020). *J. Synchrotron Rad.* **27**, 558-566.

Klementiev, K., Norén, K., Carlson, S., Clauss, K. G. V. S. & Persson, I. (2016). *J. Phys. Conf. Ser.* **712**, 012023.

Kutz, R. B., Chen, Q. M., Yang, H. Z., Sajjad, S. D., Liu, Z. C. & Masel, I. R. (2017). *Energy Technol.* **5**, 929-936.

Macrae, C. F., Sovago, I., Cottrell, S. J., Galek, P. T. A., McCabe, P., Pidcock, E., Platings, M., Shields, G. P., Stevens, J. S., Towler, M. & Wood, P. A. (2020). *J. Appl. Crystallogr.* **53**, 226-235.

Masse, R., Guitel, J. C. & Durif, A. (1979). *Acta Crystallogr.* **B35**, 1428-1429.

McMillan, J. A. (1960). *J. Inorg. Nucl. Chem.* **13**, 28-31.

Newville, M. (2013). *J. Phys. Conf. Ser.* **430**, 012007.

Niggli, P. (1922). *Zeitschrift für Kristallographie* **57**, 253-299.

Norby, P., Dinnebier, R. & Fitch, A. N. (2002). *Inorg. Chem.* **41**, 3628-3637.

Rodríguez-Carvajal, J. (1993). *Physica B Condens.* **192**, 55-69.

Sagadevan, S., Alshahateet, S. F., Anita Lett, J., Fatimah, I., Poonchi Sivasankaran, R., Kassegn Sibhatu, A., Leonard, E., Le, M.-V. & Soga, T. (2023). *Inorg. Chem. Commun.* **148**, 110288.

Salkind, A. J. & Zeek, W. C. (1959). *Journal of The Electrochemical Society* **106**, 366.

Schökel, A., Etter, M., Berghäuser, A., Horst, A., Lindackers, D., Whittle, T. A., Schmid, S., Acosta, M., Knapp, M., Ehrenberg, H. & Hinterstein, M. (2021). *J. Synchrotron Rad.* **28**, 146-157.

Standke, B. & Jansen, M. (1986). *Z. anorg. allg. Chem.* **535**, 39-46.

Standke, B. & Jansen, M. (1987). *J. Solid State Chem.* **67**, 278-284.

Stehlík, B. & Weidenthaler, P. (1959). *Collect. Czech. Chem. Commun.* **24**, 1416-1419.

Stehlik, B., Weidenthaler, P. & Vlach, J. (1959). *Collect. Czech. Chem. Commun.* **24**, 1581-1588.

Suzuki, T. (1960). *J. Phys. Soc. Jpn.* **15**, 2018-2024.

Thatcher, Z., Liu, C. H., Yang, L., McBride, B. C., Tran, G. T., Wustrow, A., Karlsen, M. A., Neilson, J. R., Ravnsbaek, D. B. & Billinge, S. J. L. (2022). *Acta Crystallogr. A* **78**, 242-248.

Vereshchagin, L. F., Popova, S. V., Serebrjanaja, N. R. & Kabalkina, S. S. (1963). *Dokl. Akad. Nauk SSSR* **152**, 853-855.

Wyckoff, R. W. G. (1922). *American Journal of Science* **3**, 184-188.

Yang, L., Culbertson, E. A., Thomas, N. K., Vuong, H. T., Kjaer, E. T. S., Jensen, K. M. O., Tucker, M. G. & Billinge, S. J. L. (2021). *Acta Crystallogr. A* **77**, 2-6.

Yang, X., Juhas, P., Farrow, C. L. & Billinge, S. J. (2014). *arXiv preprint arXiv:1402.3163*.

Yoon, Y., Yan, B. & Surendranath, Y. (2018). *J. Am. Chem. Soc.* **140**, 2397-2400.
